# Supplementary material for: Cobalt-free composite-structured cathodes with lithium-stoichiometry control for sustainable lithium-ion batteries
Source: Nat Commun. 2024 Jan 10;15:430. doi: 10.1038/s41467-023-44583-3 (PMC10782004; doi:10.1038/s41467-023-44583-3)
Supplement: Supplementary file 1 — Supplementary Information [file 41467_2023_44583_MOESM1_ESM.pdf]

Supplementary Information

Cobalt-Free Composite-Structured Cathodes with Lithium-Stoichiometry Control for Sustainable Lithium-ion Batteries

Ke Chen[1], Pallab Barai[2], Ozgenur Kahvecioglu[2], Lijun Wu[1], Krzysztof Z. Pupek[2], Mingyuan Ge[1], Lu Ma[1], Steven N. Ehrlich[1], Hui Zhong,[3] Yimei Zhu[1], Venkat Srinivasan[2], Jianming Bai[1, *], Feng Wang[1, 2, *]

1. Brookhaven National Laboratory, Upton, NY 11973, USA

2. Argonne National Laboratory, Lemont, IL 60439, USA

3. Department of Joint Photon Science Institute, Stony Brook University, Stony Brook, NY 11794, USA

Corresponding authors: JB: jmbai@bnl.gov; FW: fengwang@anl.gov

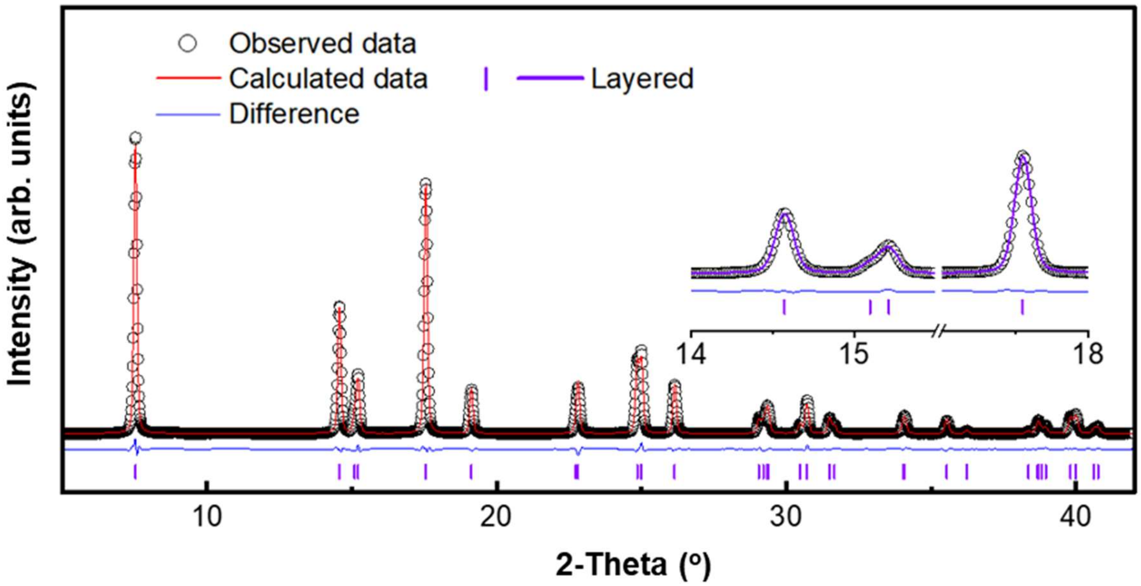

Fig S1. Synchrotron XRD patterns and Rietveld refinement results of NM9505-1.05Li, fitted with a single-phase model (layered).

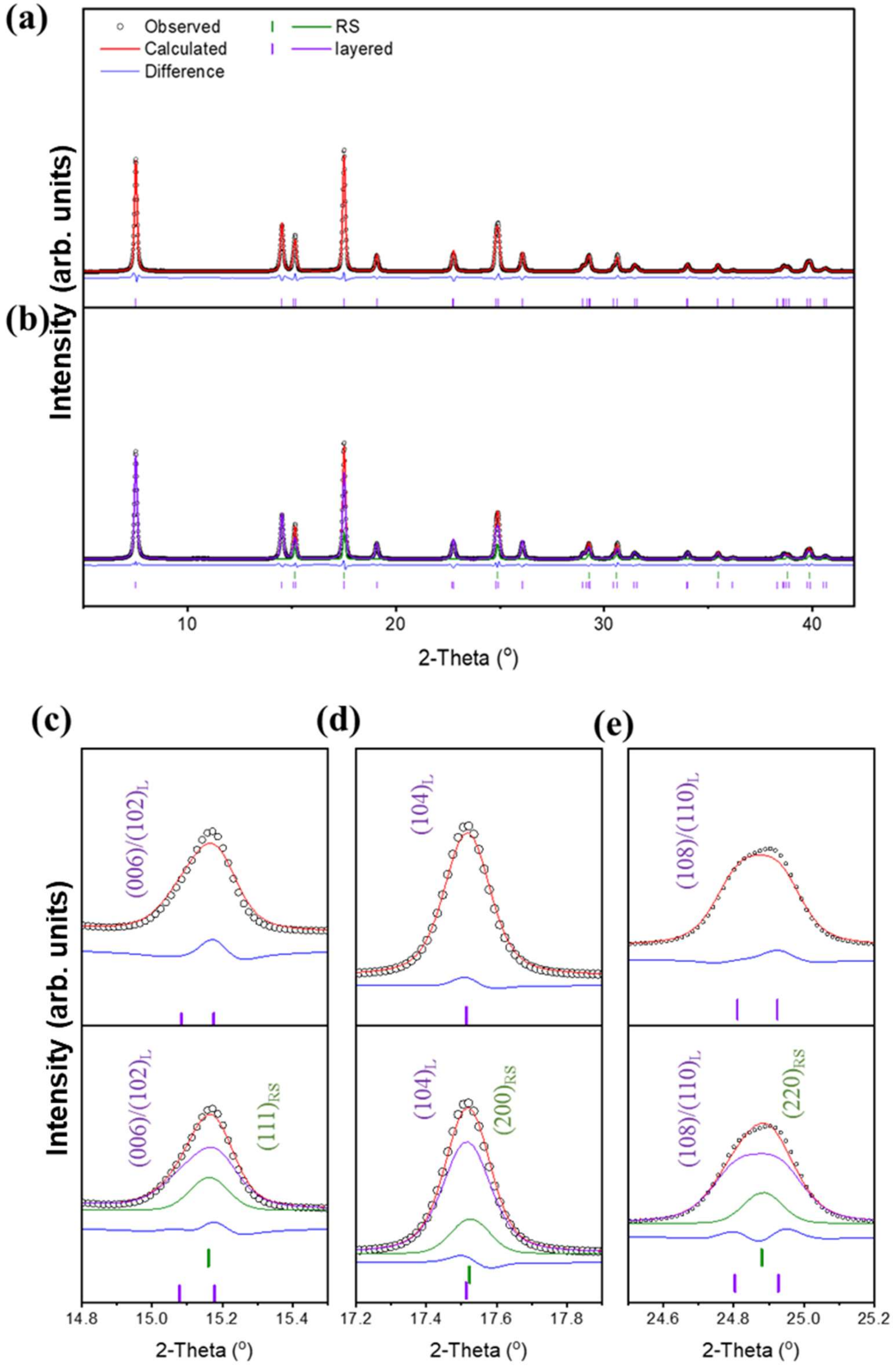

Fig. S2. Comparison of XRD fitting of NM9505-0.95Li using (a) 1-phase and (b) 2-phase models. Zoomed in view at (c) 14.8-15.5°, (d) 17.2-17.9°, and (e) 24.5-25.2°

Supplementary Note 1. Single-phase model (layered, **Figure S2a**) and two-phase model (layered and RS, **Figure 2**b) were compared in NM9505-0.95Li XRD fitting. While using the pure layered model, the Rwp of 2.83 was obtained. It can be noticed that the peaks at 15.2 (Figure S2c), 17.5 (Figure S2d), and 24.8^{o} (Figure S2e) could not be adequately matched with the pure layered phase. These peaks are related to the RS phase. By adding the RS phase to the model as shown in Figure 2b, the Rwp was improved to 2.36, indicating a much better fitting.

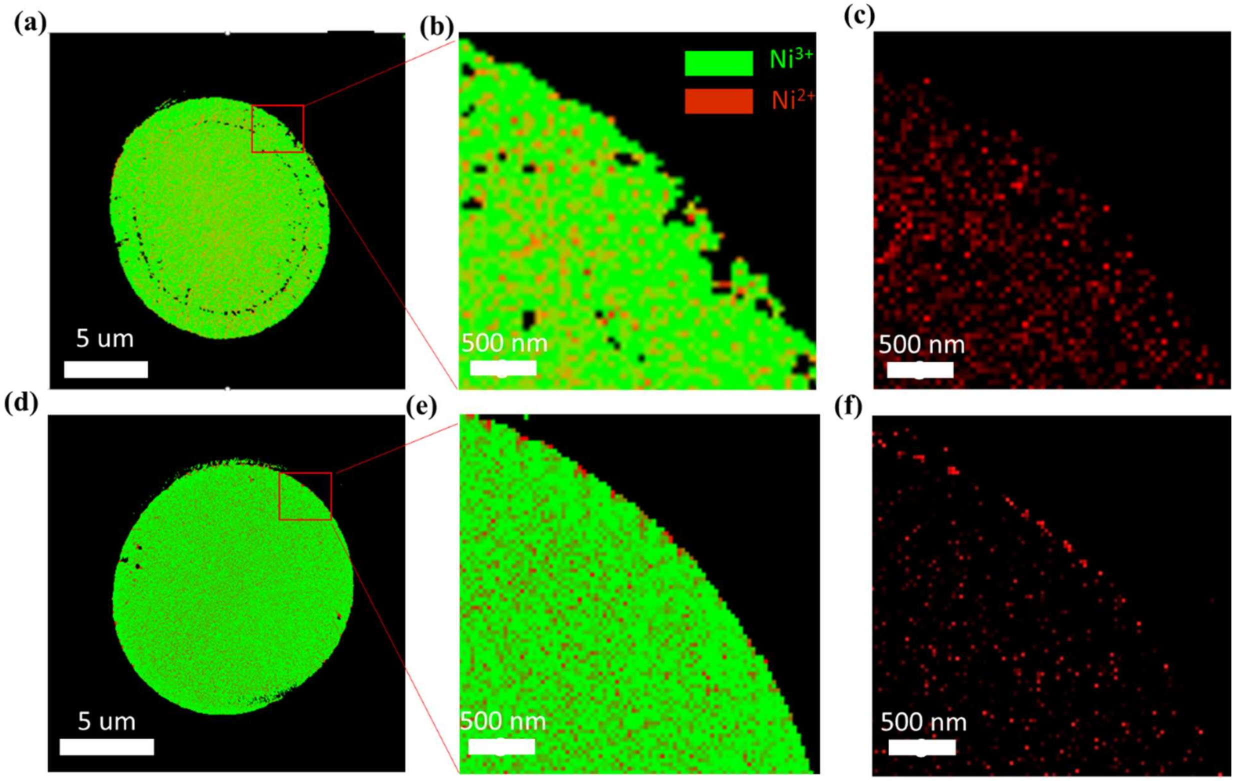

Fig. S3. **Center slice of TXM images of NM9505 -0.95Li and 1.05Li** (a)(d) The center slide of the 3D TXM mapping of NM9505-0.95Li and 1.05Li. (b) and (e) are the zoom-in views. Green and red color indicating Ni^{3+} and Ni^{2+} distribution, respectively. (c) (f) the distribution of Ni2+ of 0.95Li and 1.05Li.

Supplementary Note 2. The Supplementary Movie 1 shows the 3DTXM data of NM9505-0.95Li. Each frame in the video represents a slice of the reconstructed particles of NM9505-0.95Li with a play speed of 20 slices per second. The different colors stand for the distribution of Ni^{3+} (green) and Ni^{2+} (red). To better show the distribution, the contrast has been manually changed to highlight the Ni^{2+} (Supplementary Movie 2 and 3 shows the real intensity values). In the layered phase ($Li(Ni^{3+}{}_xNi^{2+}{}_{(0.95-x)}Mn_{0.05})O_2$), most stays in the chemical state of 3+, while in the RS (Li_y ($Ni^{3+}{}_y$ $Ni^{2+}{}_{(1-y)}$) $O_{(1+y)}$), Ni mainly stays in Ni^{2+} state. Therefore, Ni^{2+} could be used to represent the

distribution of RS. It can be clearly seen that the layered and RS phases are well-intermixed, showing an intergrown composite structure across the particles from the surface to the core.

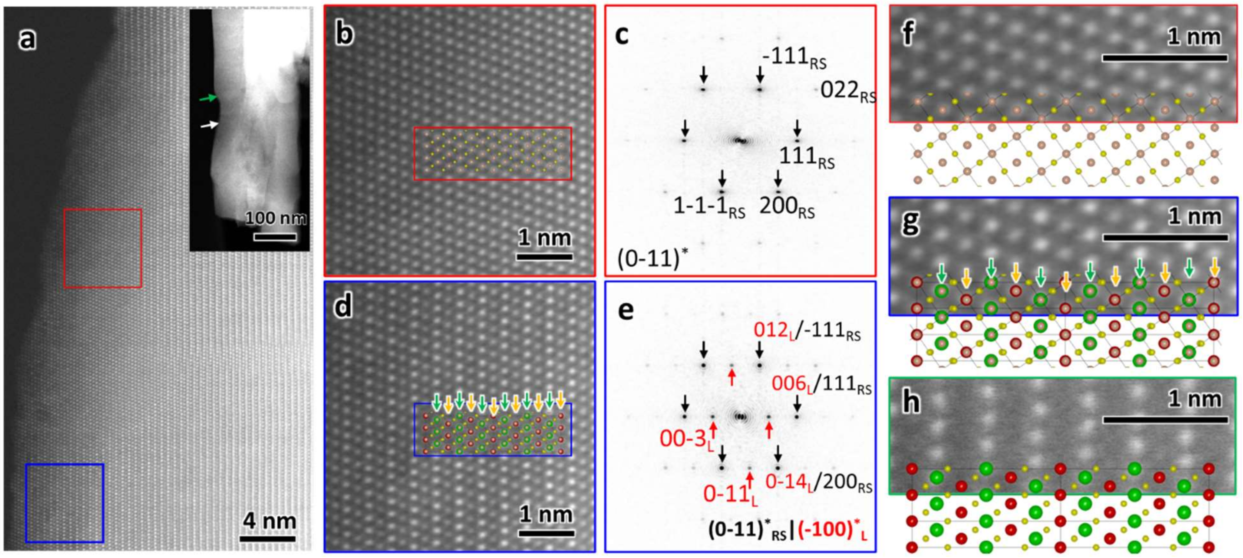

Fig. S4, **STEM images of NM9505-0.95Li.** (a) STEM-HAADF image taken viewed along [100] direction from NM9505-0.95Li. The inset shows the low magnification image where the image in (a) is taken from the area indicated by the white arrow. (b-e) Magnified images (b,d) and their corresponding FFTs (c, e) from the red and blue square in (a), showing the rock-salt and a mixture of rock-salt and layered structure, respectively. The [0-11] projection of the rock-salt and [100] projection of the layered structure are embedded in the images. Orange, yellow, red and green spheres represent Li/TM, oxygen, Li and TM, respectively. The FFTs are indexed based on RS and layered structure, respectively. Subscripts RS and L represent RS and layered structure, respectively. (f-h) Magnified images from the area marked by rectangles in (b) and (d), and the area indicated by the green arrow in (a), respectively.

Supplementary Note 3. The images in (b) and (f) show atomic columns with equal intensity, consistent with the rock-salt structure (see embedded [0-11] projection of rock-salt structure). The corresponding FFT in (c) is consistent with the (0-11)* diffraction pattern of rock-salt structure, further confirming the RS structure of this local area. The image in (h) shows the layered structure where only TM atoms show contrast. The Li and oxygen atoms are invisible due to their low atomic number in Z-contrast STEM-HAADF image (Intensity approximately is proportional to Z1.7, Z is an atomic number). The images in (d) and (g) show the layered arrangement with weak and strong contrast alternately arranged horizontally. We attribute this area to the mixture of RS and layered structures. The layered and RS structures are overlapped along the beam direction. The layers with strong contras indicated by green arrows in (g) are the overlap of TM layer (green spheres) of the layered structure with Li/TM (orange) of RS, while those with weak contrast indicated by orange arrows are the overlap of Li layer (red) of the layered structure with Li/TM (orange) of RS. In FFT (e) from this area, the spots indicated by the black arrows are contributed by both the layered structure and RS, thus showing stronger intensity than those indicated by the red arrows, which exclusively belong to the layered structure. This further confirms the mixture of layered structure and RS in this area.

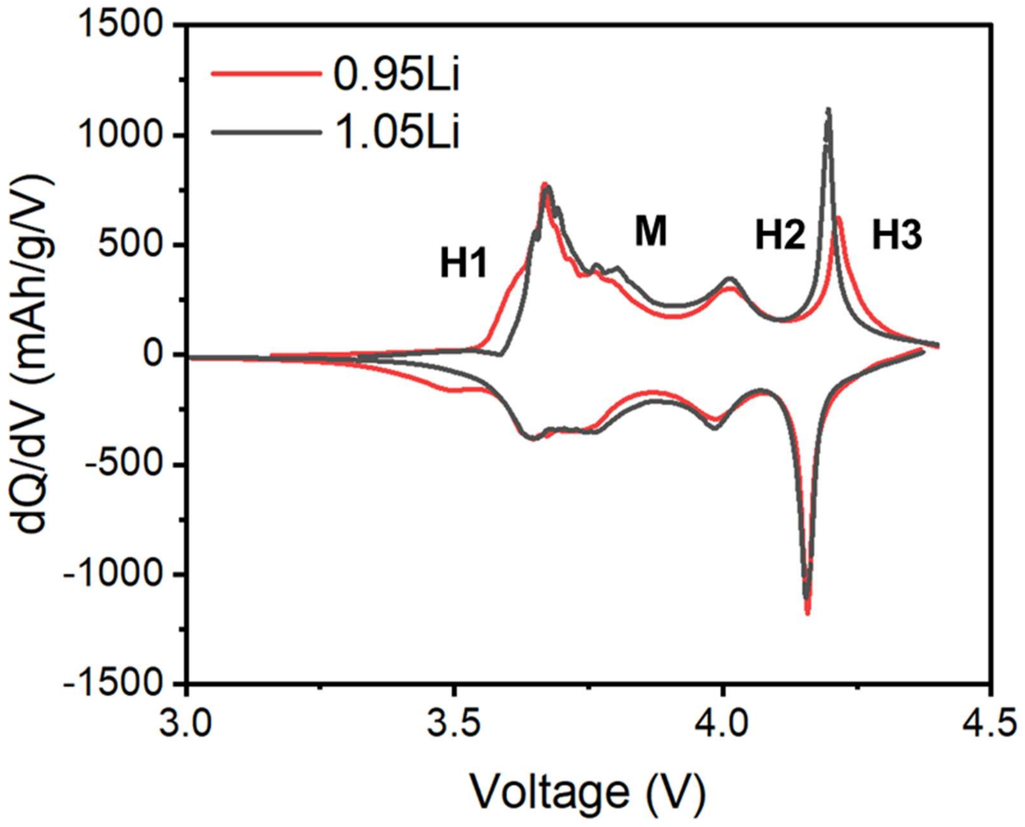

Fig. S5. The dQ/dV curves of NM9505-0.95Li and -1.05Li, showing the suppressed phase transformations, particularly the H2 to H3.

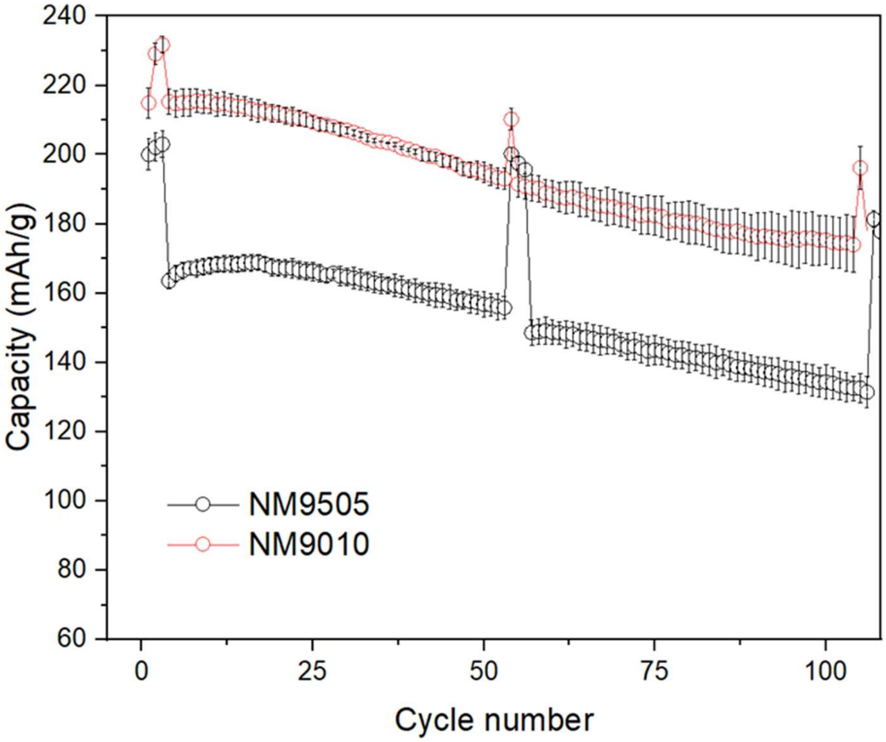

Fig. S6. Battery cycling performance of NM9505 and NM9010 at 0.5C. The error bar represents the standard deviation of three repeat cells.

Table S1. inductively coupled plasma of the NM9505 with different Li contents. Li is compared to the sum of the Transition metals.

	Li	Mn	Ni
NM9505-0.90 Li	0.940	0.048	0.952
NM9505-0.95 Li	0.979	0.048	0.952
NM9505-1.0 Li	1.024	0.048	0.952
NM9505-1.025 Li	1.049	0.048	0.952
NM9505-1.05 Li	1.077	0.048	0.952
NM9505-1.1 Li	1.128	0.048	0.952

Supplementary Note 4. Each transition metal is compared to the sum of all the Transition Metals. The measured value is slightly higher than the nominal value, which is probably due to the weighting errors of the LiOH·H2O precursors. In addition, a small portion of LiOH·H2O loses its crystal water and becomes LiOH during the storage of the materials.

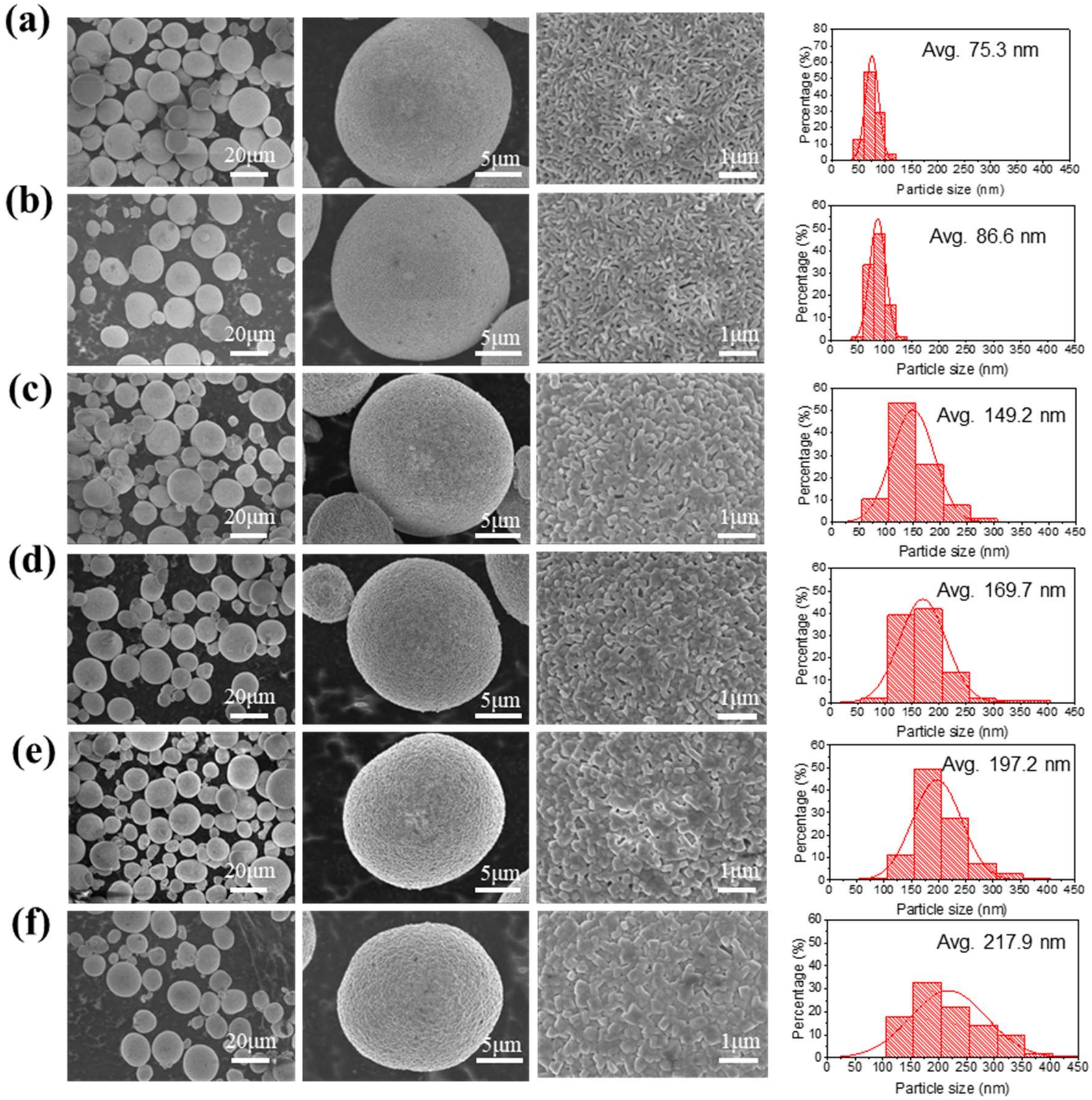

Fig. S7. SEM images of NM9505 at different magnifications. (a)-(f) NM9505 with 0.90Li, 0.95Li, 1.0Li, 1.025Li, 1.05Li and 1.1Li, and the corresponding primary particle size distribution.

Supplementary Note 5. The obtained NM9505 shows a second particle size ranging from 5 to 20 μm which seems to be not affected by the Li stoichiometry. However, the primary particle size is largely dependent on Li stoichiometry. The primary particles of NM9505 obtained with a deficient

Li source show needle-like morphology, which is inherited from the $Ni_{0.95}Mn_{0.05}(OH)_2$ precursors. Due to the RS formation, the growth of the primary particles is largely hindered. The average primary particle size increases from 75 nm to 240 nm, with increased Li contents.

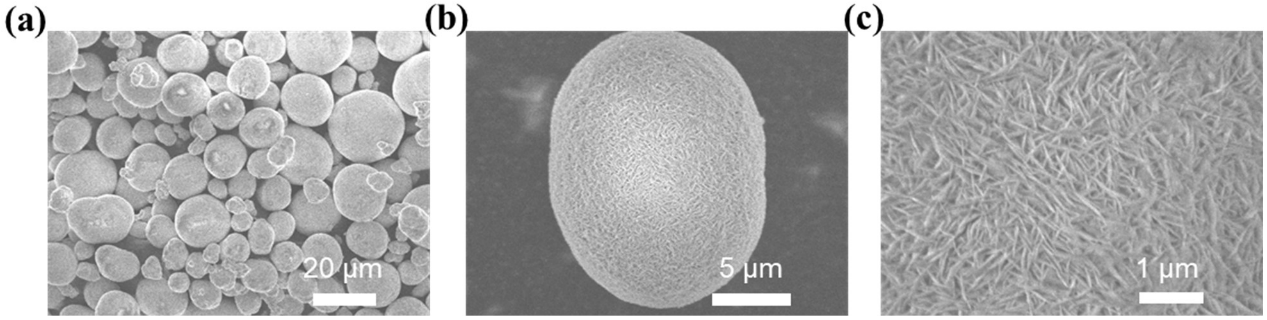

Fig. S8. SEM images of the NM9505 precursors ($Ni_{0.95}Mn_{0.05}(OH)_2$) at different magnification. (a) SEM image at low magnification, (b) NM9505 secondary particle, and (c) primary particles.

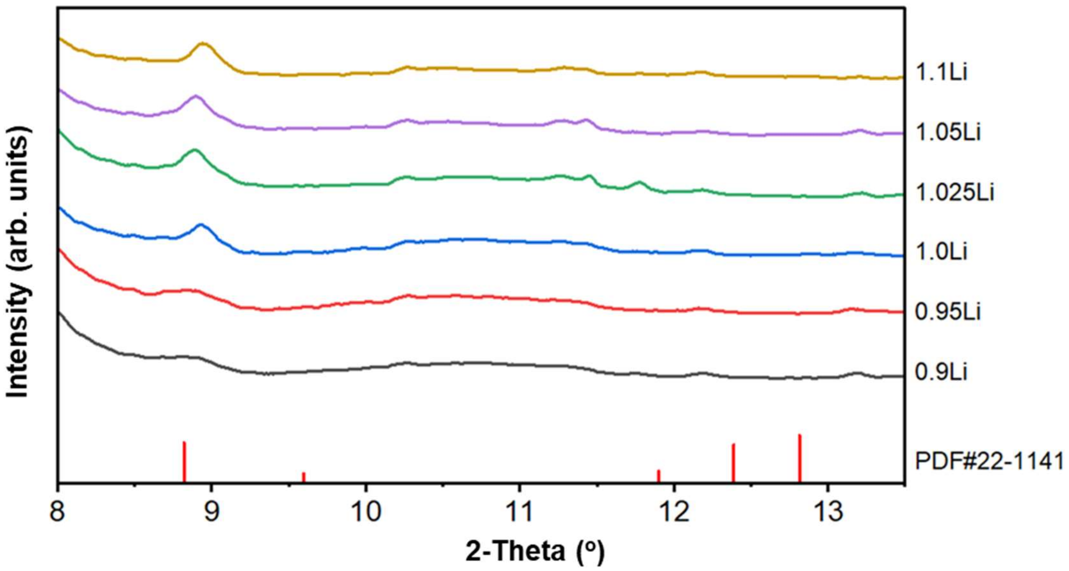

Fig. S9. Zoom-in of the XRD patterns of NM9505 with different Li contents. The higher Li content leads to the formation of Li_2CO_3 on the cathode surface.

Supplementary Note 6. The extra LiOH added during the synthesis process cannot be fully incorporated into the structure and stays at the particle surface. The residual LiOH may react with CO_2 and turn into Li_2CO_3 when exposed to the air during the sample preparation and transfer.

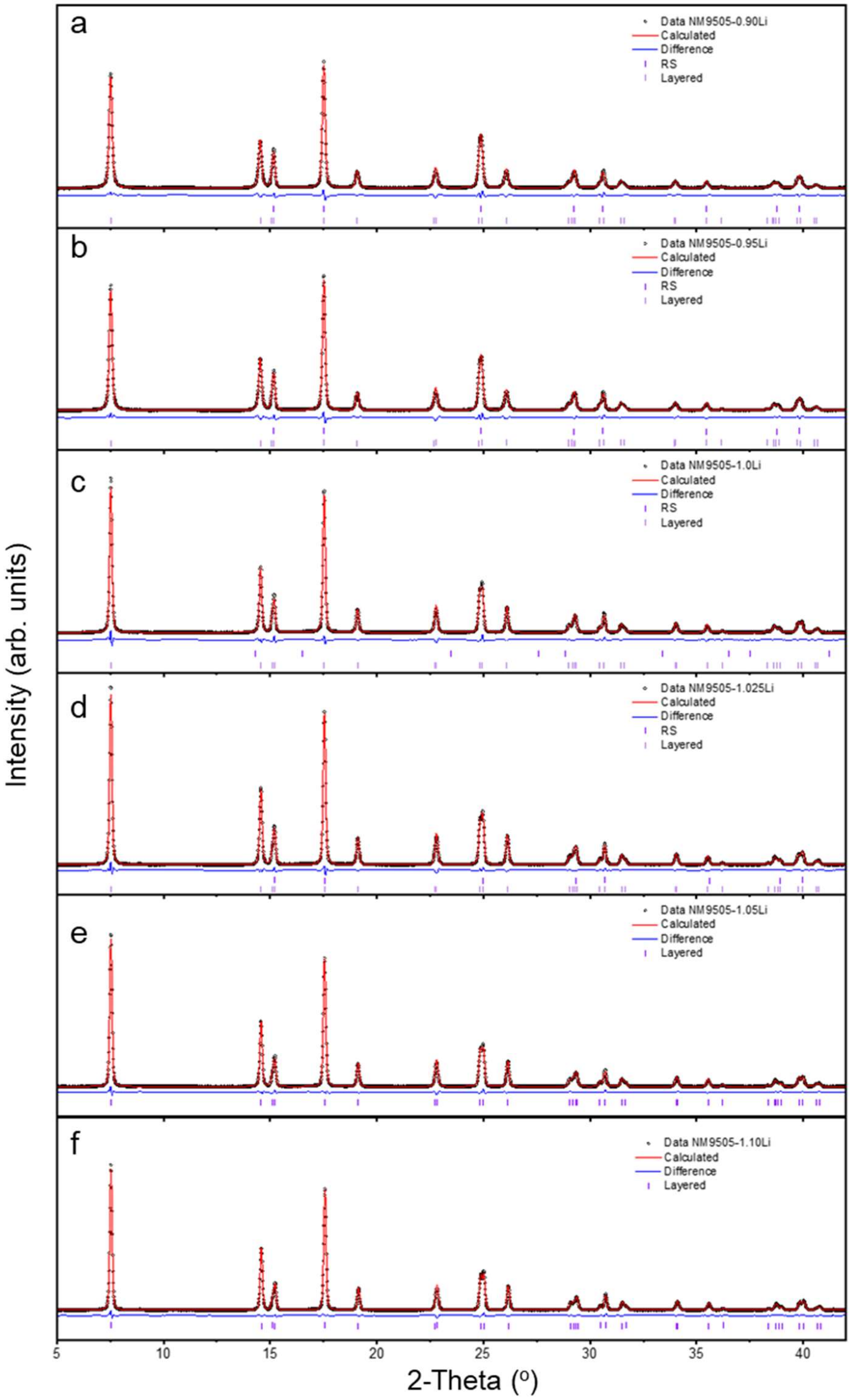

Fig. S10. Synchrotron XRD patterns (λ = 0.6199 Å) and Rietveld refinement results of (a) NM9505-0.90Li, (b) 0.95 Li, (c) 1.0Li, (d) 1.025Li, (e)1.05Li, and (f) 1.1Li.

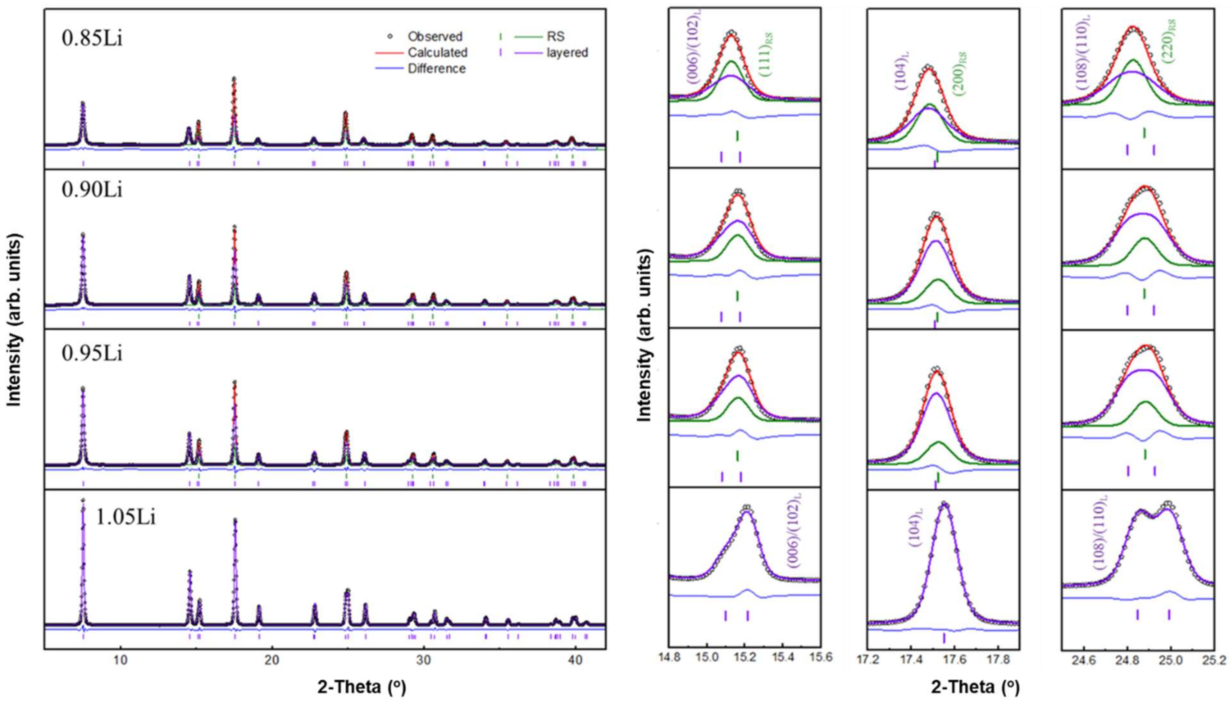

Fig. S11. Synchrotron XRD of NM9505 with 0.85Li, 0.9Li, 0.95Li and 1.05Li.

Table S2. Refinement parameters of NM9505 with different Li contents.

Li/TM add	RS-mol	a(RS)	a-L	c-L	Li-slab	Ni-mix	L-domain size	c/a	Li Occupancy (RS)	Li/TM	Rwp
0.90	0.232	4.083	2.882	14.222	2.6675	0.008	45.98	4.934	0.358	0.916	2.35
0.95	0.196	4.082	2.881	14.219	2.6617	0.021	52.09	4.934	0.359	0.906	2.35
1.00	0.033	4.074	2.878	14.211	2.6371	0.035	97.53	4.936	0.24	0.917	2.06
1.025	0.037	4.070	2.876	14.205	2.6360	0.018	99.59	4.939	0.310	0.951	2.18
1.05	0	-	2.874	14.2015	2.6337	0.026	118.74	4.941	0	0.950	2.03
1.10	0	-	2.871	14.191	2.6336	0.022	138.18	4.943	0	0.956	1.91

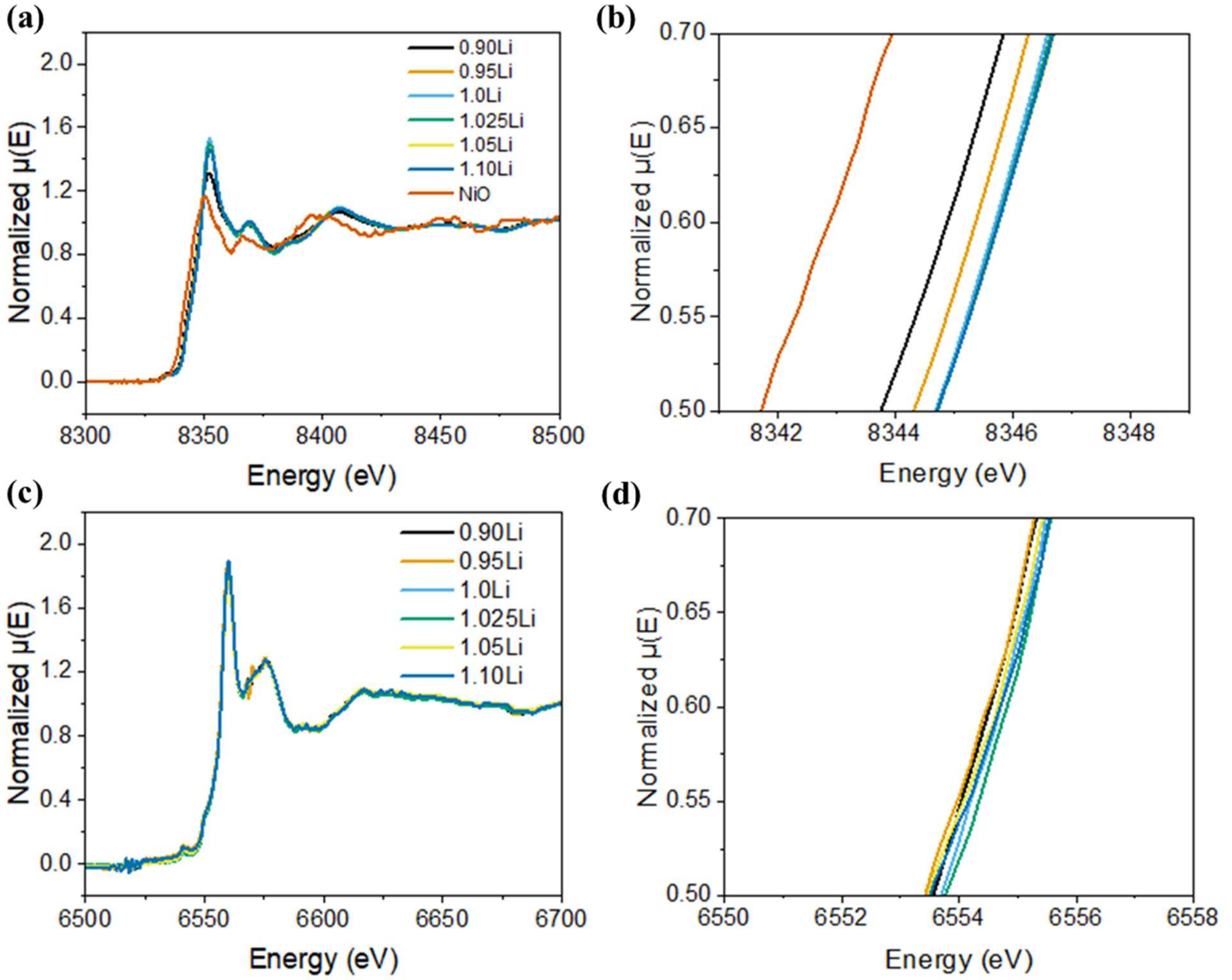

Fig. S12 X-ray absorption near-edge spectroscopy (XANES) of NM9505 obtained at different Li stoichiometry at **(a)**Ni and **(c)**Mn K-edges, and **(b)(d)** the corresponding zoom-in view as marked in red square regions.

Supplementary Note 7. At the low Li stoichiometry, with the increase of Li content, the Ni edge shift to higher energies, indicating that the average valence state of Ni increases. The results have suggested that lithiation plays a key role in Ni oxidation and the formation of the layered structure. Without enough Li source, more Ni^{2+} exists in NM9505 in the form of the RS phase. The Ni edge then keeps constant after the Li content overpasses 1.0Li, which suggests that Ni has reached its

maximum oxidation state of near 3^+ and no more Li can be incorporated into the structure. The XANES results are consistent with the XRD refinement results in that the RS phase disappears when enough Li is provided. The valence state of Mn is not significantly affected by the Li contents, as shown in **Fig. S12c** and **d**.

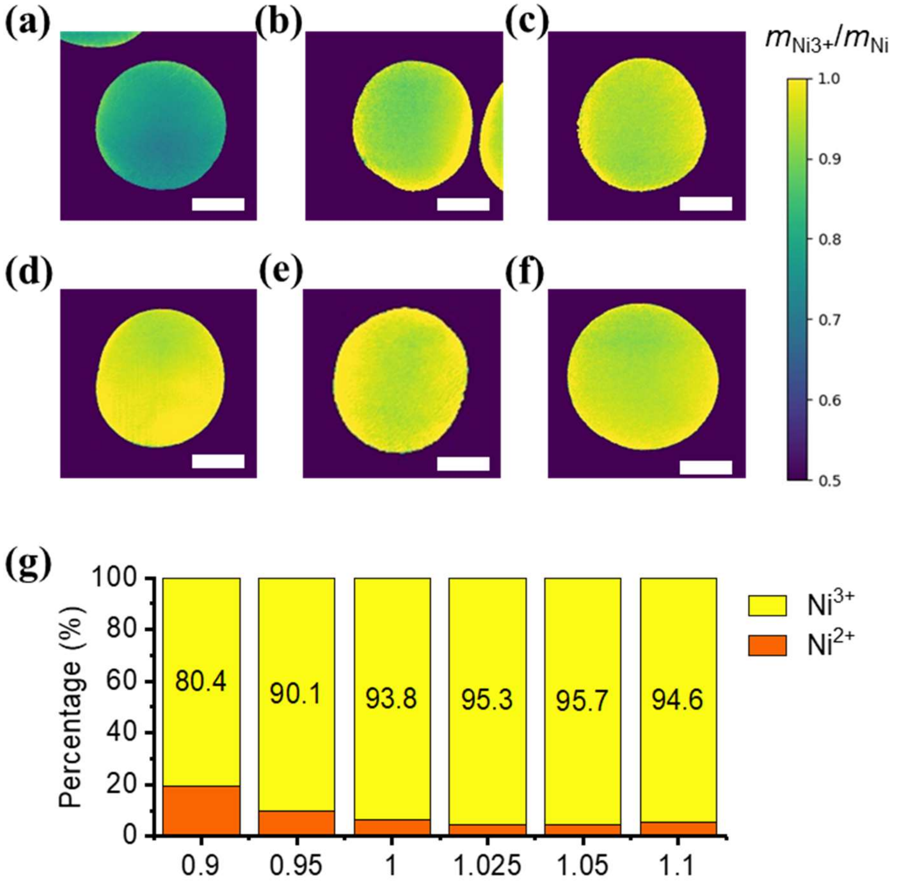

Fig. S13. 2D XANES TXM mapping of NM9505 with different Li stoichiometry of **(a)** 0.90Li, **(b)** 0.95Li, **(c)** 1.0Li, **(d)** 1.025, **(e)** 1.05Li, and **(f)** 1.10Li. The intensity stands for the intensity ratio of $Ni^{3+.}$ Scale bar is 5μm. **(g)** The quantified intensity ratio of Ni^{3+} and Ni^{2+} of the NM9505 with different Li stoichiometry.

Supplementary Note 8. X-ray absorption data shown in **Fig. S12** were obtained on bulk cathode materials, and 2DTXM XANES was used to study the distribution of Ni^{2+} and Ni^{3+} on the individual particles. The color differences in **Fig. S13** (a)-(f) stand for the concentration of Ni^{3+}. The distribution of the Ni^{3+} is uniform across the particle. As the sum of Ni^{2+} and Ni^{3+} has been set to 1, the distribution of Ni^{2+} can be reflected. The overall concentration of the Ni^{2+} and Ni^{3+}

concentration is summarized in **Fig. S13**(g), which follows the same trends in the bulk XANES measurement as shown in **Fig. S12**.

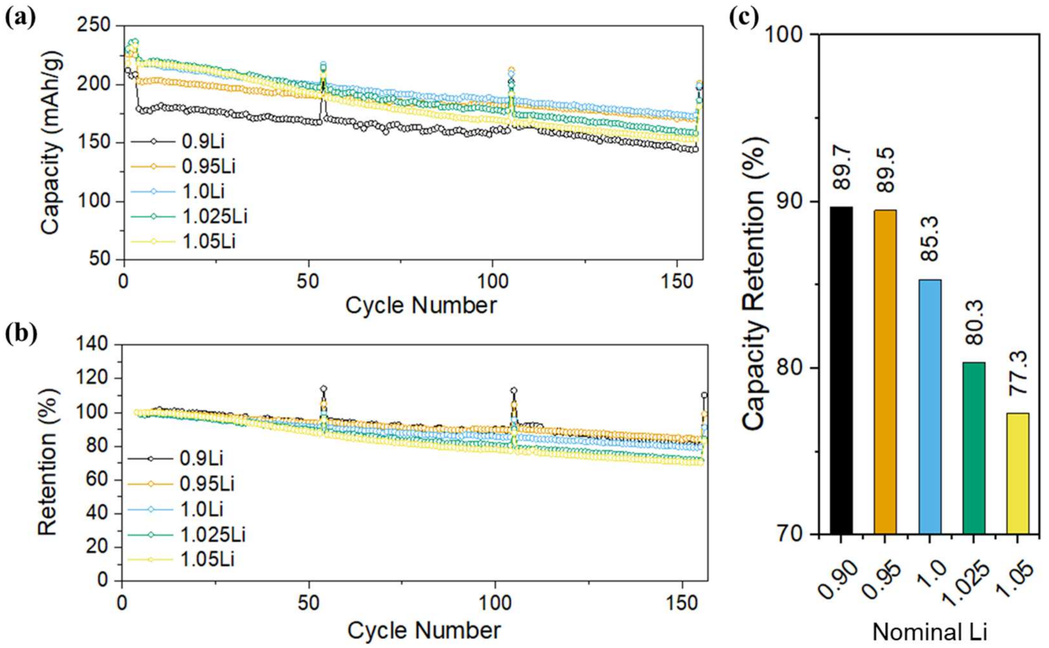

Fig. S14. **Battery cycle performance of NM9505 with different Li stoichiometry. (a)** Specific capacity and **(b)** capacity retention vs cycling number plots. (c), Capacity retention after 100 cycles.

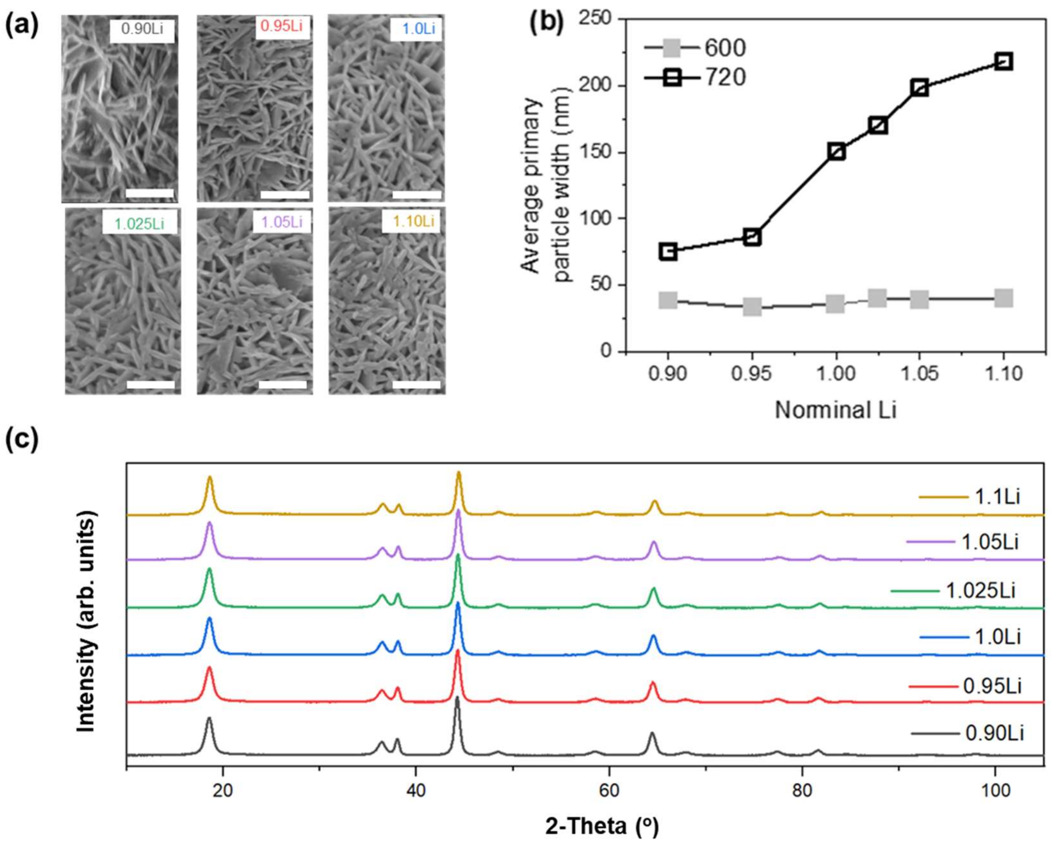

Fig. S15. Morphology and structure of NM9505 obtained at 600 ºC (a) SEM images of NM9505 with 0.90, 0.95 1.0, 1.025, and 1.05Li obtained at the temperature of 600 ºC (Scale bar 500nm). (b) Primary particle sizes of the NM9505 series with different Li stoichiometry. after calcination at 600 ºC for 12 hours (gray) and 720 ºC for 12 hours (black). **(c)** XRD patents of NM9505 obtained at 600 °C with different Li stoichiometries.

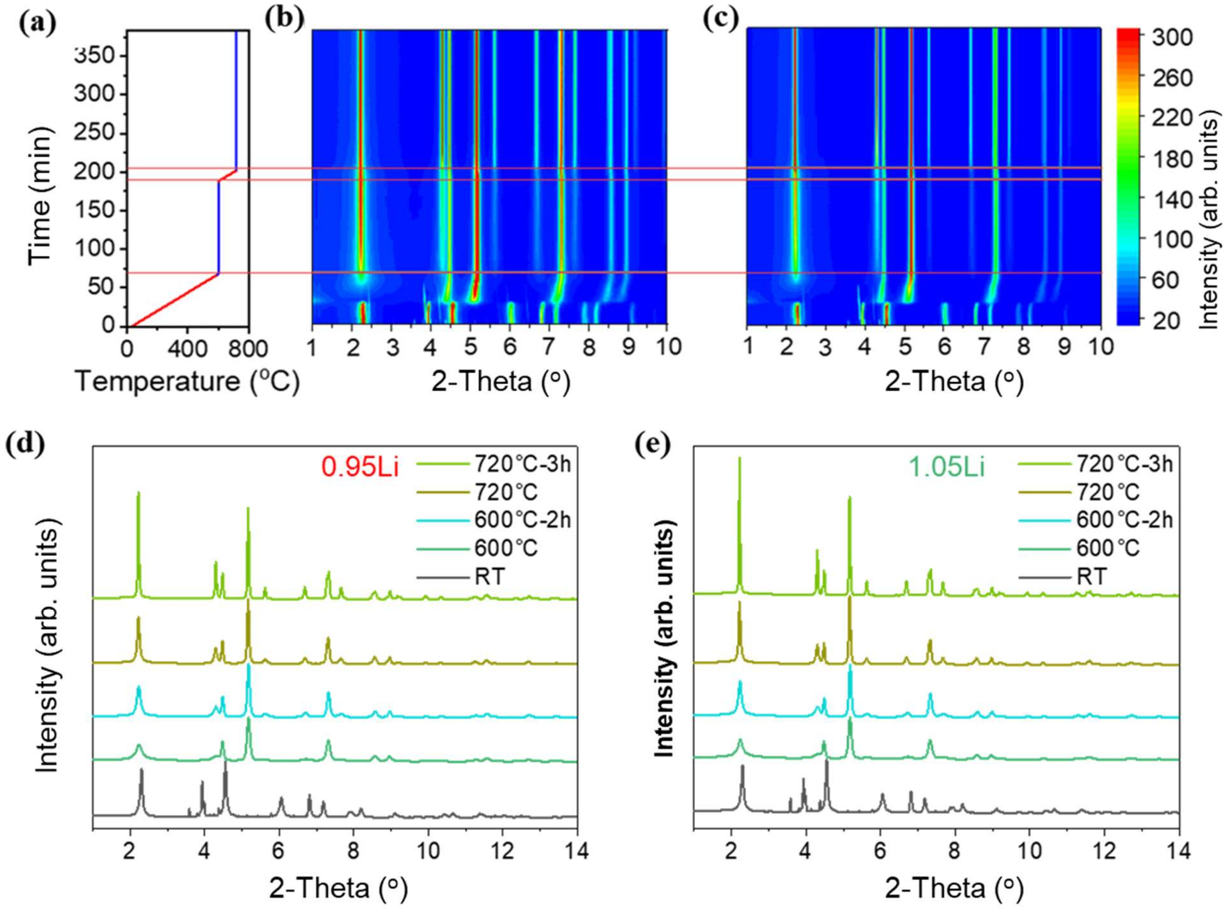

Fig. S16. In situ XRD characterization of NM9505 -0.95Li and 1.05Li (a) Heating profiles of the *in situ* synchrotron XRD experiment. (b,c) Contour plots of the time resolved in situ XRD pattens taken during calcination of NM9505-0.95Li and 1.05Li. *In situ* XRD patterns of **(d)** NM9505-0.95Li and **(e)** 1.05Li at representative temperatures of room temperature, 600 °C, 600 °C 2 hour, 720°C, and 720°C 3h.

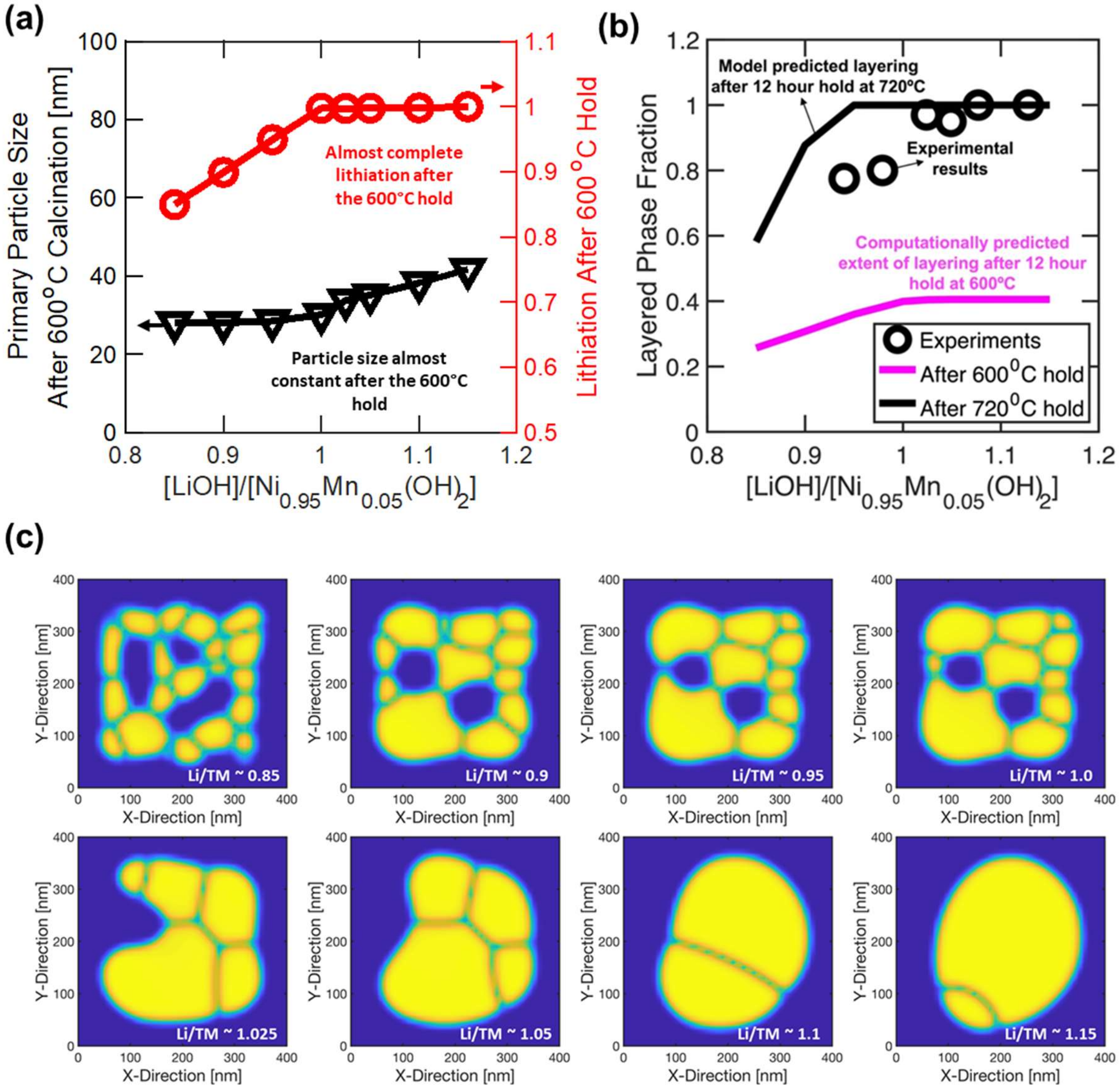

Fig. S17. Particle size evolution through modeling methods. (a) Computationally predicted cathode primary particle size (left axis and black triangles) and extent of lithiation (right axis and red circles) obtained after the 600 °C hold as a function of the Li/TM ratio. (b) Comparison between the experimentally observed and computationally predicted layered phases during the calcination process with different Li/TM ratio. (c) The computational cathode primary particle microstructure after calcination at 720 °C for 12 hours conducted at Li/TM ratio of 0.85, 0.9, 0.95, 1.0,1.025, 1.05, 1.1, and 1.15, respectively.

Supplementary Note 9.

The calcination of NM9505 is simulated at the mesoscale level using the developed computational framework that captures the following physical phenomena (Details of the simulations and used techniques are provided in the **Supplementary Note 10** (SI) section):

a) Removal of water and corresponding volume shrinkage of the cathode particles;

b) Reaction with oxygen and oxidation of the transition metals along with corresponding volume expansion;

c) Reaction with lithium salt, lithiation of the particles, and subsequent volume expansion;

d) Conversion from the lithiated rocksalt to the layered phase;

e) Surface, grain-boundary, and bulk diffusion mediated sintering of the cathode particles;

f) "Liquid phase sintering", which is modeled through an enhanced magnitude of the surface and the grain-boundary diffusivities.

Different physical phenomena become dominant at different temperatures. For example, removal of water from the transition metal hydroxides is dominant at temperatures lower than 300°C, whereas oxidation and reaction with the lithium salt becomes dominant only at temperatures higher than 450°C (because the lithium salt, LiOH, melts at around that temperature). Also note that conversion from the rocksalt to the layered phase occurs mostly beyond 600°C. All the parameters that govern the reaction kinetics are determined from the experimentally observed evolution of different phases. Similarly, the "liquid phase sintering" is only invoked if excess lithium salt is introduced during the calcination process.

Computationally predicted extent of lithiation after the 600°C hold (red circles along the right axis in Figure S17(a)) indicates that almost all the lithium salt reacts with the transition metal oxides and intercalates into the cathode particles within 600°C, as long as it does not violate the stoichiometry of the final cathode $\left((c_{\mathrm{Li}}/c_{\mathrm{Li,max}}) \leq 1\right)$. Note that even though complete lithiation of the cathode particles occur after the 600°C hold, the lithiated cathodes remain in a disordered rocksalt phase, and does not immediately convert into a layered structure. The solid magenta line in Figure S17 (b) clearly indicates that partial evolution of the layered phase after the 600°C hold is predicted by the computational model. Evolution of the layered phase is assumed to occur through nucleation and growth of the layered domains within a disordered rocksalt matrix when the lithium stoichiometry exceeds a certain threshold of 0.85 $\left((c_{\mathrm{Li}}/c_{\mathrm{Li,max}}) \geq 0.85\right)$. The growth process of these layered regions is assumed to be a rate-limiting step, which becomes dominant only during the high temperature hold at 720°C. The black solid line in Figure S14 (b) indicates the extent of layered phase evolution predicted by the computational model after the 720°C hold, which shows complete layering of the cathode particles with close to, or greater than, stoichiometric amount of lithium $\left((\mathrm{Li}/\mathrm{TM}) \gtrsim 1\right)$. The experimentally observed extent of layered phase formation after the 720°C hold is shown by the black circles, which indicates a qualitative correlation with computational predictions. Even with a close to the stoichiometric amount of lithium $\left((\mathrm{Li}/\mathrm{TM}) \sim 0.95\right)$, only 80% layering of the cathode particles is observed experimentally. However, the computational model predicts almost 100% evolution of the layered phase. This mismatch between the theory and experiment, as observed in Figure S14, can possibly be attributed to two different features: a) The computational mode assumes faster reaction rate kinetics for the transformation from rocksalt to the layered phase; b) Mixing of lithium and transition metal ions

are not taken into consideration in the computational model, which is extensively observed in cathodes during calcination. Also note that, in Fig. S17(b), even if excess lithium salt is added during the calcination process $((\mathrm{Li/TM}) > 1)$, the extent of lithiation cannot exceed unity because lithium-rich cathodes are not being considered in the present context.

During calcination at lower temperatures (<600 ℃), the variations in the particle size (decrease due to the removal of water and increase because of oxidation and lithiation processes) can be considered as a thermodynamic phenomenon.[1] Whereas the diffusion induced increase in particle size occurs at high temperatures, which is also known as the sintering process, and can be categorized as a kinetic phenomenon.[2, 3] There can be several factors influencing the diffusion of mass within the particles of CAMs:[3, 4]

i. Calcining temperature, where increasing temperature results in enhanced diffusivity and faster transport of Li and TM species.
ii. Lattice structure of the CAMs, where Li and TM species within the well-defined layered structure should demonstrate faster diffusion compared to that within the disordered rocksalt, or spinel phases where TM and Li ions are intermixed with each other.
iii. Presence of sintering aids, where the existence of an extra liquid domain adjacent to the particles facilitates the rearrangement of Li and TM species, thereby enhancing their diffusion through the liquid region.

By considering all these factors, the effects of calcining conditions (such as temperature and Li/TM ratio) on the size and size distribution of the primary particles in the final CAMs are simulated through computational modeling. The NM9505 hydroxide precursor particle microstructure, consisting of spherical particles, is computationally generated in two dimensions (2D) with an

average particle diameter of around 35 nm, which is shown in Fig. 4(a) (Main text). The size of these circular particles is assumed to be equivalent to the smaller dimension of the experimentally observed NM9505-hydroxides because diffusion-induced variations mostly occur along the shorter dimension. The computationally generated particle microstructures after calcination were subjected to a very similar temperature profile to the experimental one with 12 h temperature holding at 600 ℃ and another 12 h at 720 ℃.

Particle morphology after holding at 600 ℃ exhibits an average primary particle size similar to that of the initial microstructure despite the dehydration, oxidation, lithiation, and sintering processes. It is almost independent of the amount of lithium (Li/TM ratio) added during the calcination process, with the same magnitude as the precursors (Fig. S17a). This lack of increase in particle size at 600 ℃ can be attributed to the very slow diffusion kinetics at low temperatures.[5-7] In addition, the lithiation-induced volume expansion is compensated by the dehydration-induced volume shrinkage.[1] With lithium stoichiometry greater than unity (Li/TM >1), the slight increase in particle size with increasing lithium content can be attributed to the initiation of the "liquid phase sintering" process, which will be further discussed in detail below.

Substantial growth of the primary particles, with strong dependence on the Li/TM ratio, is observed after the 12 h holding at 720 ℃, (Fig. S17c). It is evident that the rate of increase in particle size is low for Li/TM ratio less than unity (Li/TM<1), while a significantly larger rate of increase is observed at more than stoichiometric amount of Li (Li/TM>1). The qualitative correlation between theory and experiment indicates that the various physicochemical phenomena that occur during the calcination of CAMs at elevated temperatures are well captured by the developed computational framework. Based on experimental and simulation results, primary

particle size is largely dependent on the Li/TM ratios in final CAMs, with other synthesis conditions remaining the same. We proposed that primary particle growth is mainly mediated by mass transfer, which is predominantly determined by two mechanisms, namely lithiation-induced crystallization and liquid phase sintering.

For less than a stoichiometric amount of lithium (Li/TM<1), both RS and layered phase exists, showing different lattice structures. Li and TM ions are mixed with each other in the RS phase, and it is hypothesized that the RS phase demonstrates lower magnitudes of diffusion coefficient for the mass transport process. In contrast, the layered phase demonstrates higher diffusivities due to their ease of sliding with respect to each other, where the Li and TM ions exist in distinct layers. This hypothesis stems from the observation that lithium diffusion is much faster in the layered phase as compared to the disordered RS one.[8, 9] Accordingly, in the developed computational model, larger magnitudes of surface, grain-boundary, and bulk diffusivity of the primary particles are assumed for the layered phase as compared to the RS. The effective increase in cathode primary particle size with increasing lithium content, while still maintaining less than a stoichiometric amount of lithium (Li/TM <1), is defined as "lithiation-induced crystallization". Therefore, in the region of Li deficiency, a higher Li/TM ratio leads to a slightly larger primary particle size.

For calcination with excess lithium salt (Li/TM >1), all the primary particles experience complete layering irrespective of the amount of lithium (Fig. S17 (b)), and they demonstrate a very similar extent of "lithiation-induced crystallization". However, experimentally observed primary particles keep increasing in size with lithium content even for more than stoichiometric amounts of Li. It is worth noting that even if more than a stoichiometric amount of lithium is added before the calcination process, only the stoichiometric amount of lithium reacts with the primary particles. The excess lithium salt, LiOH in the present case, sits around the particles in a molten state because

the calcination temperature (720 ℃) is much higher than the melting point of LiOH (around 462 ℃). These excess lithium salts in liquid state help to further sinter the particles through the "liquid phase sintering" mechanism.[10] Faster mass transport through the liquid phase is observed, which helps in the sintering of the particles.[11] Since the liquid phase is only observed near the surface and grain boundaries of the primary particles, the magnitude of only the surface and grain-boundary diffusion coefficients are assumed to increase through the "liquid phase sintering" process. Incorporation of this additional "liquid phase sintering" mechanism within the computational framework allows capturing the continuous increase in primary particle size with increasing lithium content when excess lithium salt is introduced during the calcination process.

Supplementary Note 10 Computational Methodology

In order to provide a better understanding of the calcination mechanism of the NiMn9505$(OH)_2$ precursors, a phase field based continuum model has been developed. Various chemical reactions (dehydration, oxidation, lithiation, layering, etc.) and sintering processes (mediated by surface, grain-boundary, and bulk diffusion mechanisms), occurring at high temperature, are modeled within the continuum framework by solving appropriate partial differential equations. The different physicochemical phenomena, and the corresponding partial differential equations, being simulated in the computational methodology are described below in detail.

Sintering process: For capturing the sintering of NiMn9505 particles, a combination of Chan-Hilliard (for modeling the conserved phase parameters) and Allen-Cahn type equations (for

modeling the non-conserved phase parameters) are solved in the phase-field framework. Following previously published techniques, a phase parameter (ρ) has been used to differentiate between the solid phase $(\rho = 1)$ and the voids $(\rho = 0)$.[12] This particular phase parameter (ρ) is a conserved one, and its evolution with time has been modeled using the Cahn-Hilliard equation:[12, 13]

$$\frac{\partial \rho}{\partial t} = \vec{\nabla} \cdot \left(D \vec{\nabla} \frac{\delta F_\mathrm{e}}{\delta \rho} \right) - \vec{\nabla} \cdot (\rho \vec{v}) \quad (1)$$

Here, t indicates time, $\vec{\nabla}$ is the gradient operator, D is the local diffusion coefficient, which depends on location, F_e indicates the total free energy, and $\vec{v}$ is the velocity vector associated with the bulk motion of the grains. Detailed expression of D and F_e will be provided later. The exact values of $\vec{v}$ are estimated from the lithiation induced volume expansion of the solid phase, which are calculated by solving the mechanical equilibrium equation explained later. Since the phase parameter ρ only differentiates between the solid and void phases, it cannot predict the evolution of different grains that participate in the sintering process. To capture the various grains, another set of phase parameter have been introduced, which is defined as η_α.[14, 15] Here, $\alpha = 1,2,3, \dots, N$, where N indicates the total number of grains within the computational domain. For grain k, $\eta_\mathrm{k} = 1$, and all other $\eta_{\alpha'} = 0$, where, $\alpha' = 1,2, \dots, (\mathrm{k}-1), (\mathrm{k}+1), \dots, N$, and $\alpha' \neq \mathrm{k}$. Since η_α's are non-conserved parameters, the Allen-Cahn equation has been used to capture their evolution with time:[12, 13]

$$\frac{\partial \eta_\alpha}{\partial t} = -L \frac{\delta F_\mathrm{e}}{\delta \eta_\alpha} - \vec{\nabla} \cdot (\eta_\alpha \vec{v}) \quad (2)$$

where, L is the order parameter scalar mobility, and the rest of the variables have been defined earlier.

The total free energy depends on the bulk chemical free energy $\left(f(\rho,\eta_\alpha)\right)$ and the gradient free energy components, which is defined as:[12, 13]

$$F_{\mathrm{e}} = \int \left[f(\rho,\eta_\alpha) + \frac{1}{2}\beta_\rho\left|\vec{\nabla}\rho\right|^2 + \sum_\alpha \frac{1}{2}\beta_\eta\left|\vec{\nabla}\eta_\alpha\right|^2\right] dv \tag{3}$$

Here, dv indicates infinitesimal volume element of the system, and β_ρ and β_η are gradient energy coefficients. The bulk chemical free energy depends on both the conserved and non-conserved phase parameters, ρ and η_α, and can be defined as:[12, 13]

$$f(\rho,\eta_\alpha) = A\rho^2(1-\rho)^2 + B[\rho^2 + 6(1-\rho)\sum_\alpha \eta_\alpha^2 - 4(2-\rho)\sum_\alpha \eta_\alpha^3 + 3(\sum_\alpha \eta_\alpha^2)^2] \tag{4}$$

Here, A and B are constants, and their magnitude depends on the grain-boundary energy (γ_{GB}) and surface energy (γ_{sf}) of the corresponding material. It is possible to write the magnitude of β_ρ, β_η, A and B in terms of γ_{GB}, γ_{sf} and interfacial width (l) as shown below:[13, 16]

$$A = \frac{12\ {}_{\mathrm{sf}} - 7\gamma_{\mathrm{GB}}}{l} \tag{5}$$

$$B = \frac{\gamma_{\mathrm{GB}}}{l} \tag{6}$$

$$\beta_\rho = \frac{3l}{4}(2\gamma_{\mathrm{sf}} - \gamma_{\mathrm{GB}}) \tag{7}$$

$$\beta_\eta = \frac{3}{4}\gamma_{\mathrm{GB}}l \tag{8}$$

The order parameter scalar mobility (L) can be written in terms of the grain boundary mobility (M_{GB}), and the interfacial width (l) as,[13]

$$L = \frac{4M_{\mathrm{GB}}}{3l} \tag{9}$$

Magnitudes of the grain-boundary energy (γ_{GB}), surface energy (γ_{sf}), grain boundary mobility (M_{GB}), and interfacial width (l) have been provided in Table: I.

The diffusion coefficient (D) depends on both the phase parameters, ρ and η_{α}, at any particular location, and is defined as:[12]

$$D = D_{\mathrm{v}}\phi(\rho) + (D_{\mathrm{s}} + D_{\mathrm{LPS}})\rho^2(1-\rho^2) + (D_{\mathrm{GB}} + D_{\mathrm{LPS}})\sum_{\alpha}\sum_{\alpha'\neq\alpha}\eta_{\alpha}\eta_{\alpha'} \tag{10}$$

where, D_{v} is the diffusion coefficient in the bulk, D_{s} is the surface diffusivity, D_{GB} indicates the diffusion coefficient within the grain-boundary domain, and D_{LPS} denotes the enhancement in the surface and grain-boundary diffusivities due to the "liquid phase sintering" mechanism in the presence of molten lithium salt. The term $\phi(\rho)$ is a polynomial in terms of ρ, which takes a value of 1.0 when $\rho = 1$, and becomes 0.0 when $\rho = 0$. The exact form of $\phi(\rho)$ used in the present analysis has been adopted from literature:[12]

$$\phi(\rho) = \rho^3(10 - 15\rho + 6\rho^2) \tag{11}$$

Using Eq. (9) and (10) different diffusion coefficients in the bulk, surface region, and grain boundaries have been implemented without explicitly tracking the individual domains.

Since calcination and sintering of LLZO is conducted at elevated temperatures, it is very important to appropriately capture the temperature dependent variation of diffusion coefficients ($D_{\mathrm{v}}, D_{\mathrm{s}}, D_{\mathrm{GB}}, D_{\mathrm{LPS}}$) and grain boundary mobility (M_{GB}). An Arrhenius type relation have been adopted to predict the change in transport properties with increasing temperature, which is written as:[13, 17]

$$D = D_0 \exp\left(-\frac{E_{\mathrm{a}}}{R_{\mathrm{g}}T}\right) \tag{12}$$

Here, D_0 indicates the pre-exponential factor, E_a is the activation energy, R_g and T denotes the universal gas constant and the temperature in Kelvin scale, respectively. Also, different values of D_0 have been adopted for bulk, surface, grain-boundary, and liquid phase sintering induced diffusion coefficients $\left(D_{\mathrm{v},0} \neq D_{\mathrm{s},0} \neq D_{\mathrm{GB},0} \neq D_{\mathrm{LPS},0}\right)$.[12] However, the activation energies for all the diffusivities and grain boundary mobility has been assumed to be the same, which changes with the chemical composition of the material.[13] Following existing literatures, the surface diffusion coefficient has been assumed to be 10 times larger than the grain-boundary diffusion $\left(D_{\mathrm{s},0} \sim 10 D_{\mathrm{GB},0}\right)$, grain boundary diffusivity is around 40 times larger than the bulk diffusivity $\left(D_{\mathrm{GB},0} \sim 40 D_{\mathrm{v},0}\right)$, and the pre-exponential factor for the liquid phase sintering induced diffusivity is assumed to be two orders of magnitude larger than the surface diffusivity $\left(D_{\mathrm{LPS},0} \sim 10 D_{\mathrm{s},0}\right)$.[12, 17] Because of the extremely small size of the computational domain (< 500 nm) as compared to the size of the pellet or the capillary (~ 1 cm), all spatial locations have been assumed to demonstrate the same temperature.[13]

The activation energy (E_a) and the pre-exponential factor of diffusivity (D_0) is assumed to be functions of oxygen $\left(c_{\mathrm{O}_2}\right)$ and lithium (c_{Li}) concentration, and the extent of layering $(\tilde{c}_\mathrm{L})$ that occur within the cathode active particles (note that the extent of layering is defined in the form of fractions). The assumed mathematical relations are provided below:

$$E_\mathrm{a} = E_{\mathrm{a},0} + \left(c_{\mathrm{O}_2}/c_{\mathrm{O}_2,\mathrm{max}}\right) \cdot E_{\mathrm{a},\mathrm{O}_2} + \left(c_{\mathrm{Li}}/c_{\mathrm{Li},\mathrm{max}}\right) \cdot E_{\mathrm{a},\mathrm{Li}} - \tilde{c}_\mathrm{L} \cdot E_{\mathrm{a},\mathrm{L}} \quad (13)$$

$$D_0 = D_{0,0} \cdot D_{0,\mathrm{O}_2}^{\left(c_{\mathrm{O}_2}/c_{\mathrm{O}_2,\mathrm{max}}\right)} \cdot D_{0,\mathrm{Li}}^{\left(c_{\mathrm{Li}}/c_{\mathrm{Li},\mathrm{max}}\right)} \cdot D_{0,\mathrm{L}}^{\tilde{c}_\mathrm{L}} \quad (14)$$

Here, $E_{\mathrm{a},0}$, $E_{\mathrm{a},\mathrm{O}_2}$, $E_{\mathrm{a},\mathrm{Li}}$ and $E_{\mathrm{a},\mathrm{L}}$ are the activation energies for sintering associated with the basic rocksalt material, oxygenated material, the lithiated particles in disordered rocksalt phase, and the

particles that exist in layered phase, respectively, whereas the D_0's are the corresponding pre-exponential factors of diffusion. Also, $c_{O_2,\mathrm{max}}$ and $c_{\mathrm{Li,max}}$ are the maximum concentrations of oxygen and lithium within the NiMn9505 particles. All the parameters used for this analysis is listed in Table S1 provided below.

Removal of water (the dehydration process): At the beginning of the calcination process, with increasing temperature water molecules from the transition metal hydroxides are expelled into the environment, which causes a shrinkage in the lattice volume. Note that during this entire dehydration process, the transition metals do not oxidize or reduce, and exist consistently in an oxidation state of 2+. The reaction of water removal occurs at the particle surface, and continuous diffusion of water molecules from the interior of the particles to the surface helps to continue the dehydration reaction. In order to simulate the diffusion of water from the particle interior to the surface, the following species reaction and transport relations are solved computationally:

$$\frac{\partial \tilde{c}_{\mathrm{H_2O}}}{\partial t} = \vec{\nabla} \cdot \left(D_{\mathrm{H_2O}} \vec{\nabla} \tilde{c}_{\mathrm{H_2O}}\right) - \vec{\nabla} \cdot \left(\tilde{c}_{\mathrm{H_2O}} \vec{v}\right) + R_{\mathrm{H_2O}} \tag{15}$$

Here, $\tilde{c}_{\mathrm{H_2O}}\left(= c_{\mathrm{H_2O}}/c_{\mathrm{H_2O,max}}\right)$ is the normalized water concentration within the cathode precursors, $D_{\mathrm{H_2O}}$ is the coefficient of diffusivity for single phase diffusion of water molecules within the cathode precursors, $\vec{v}$ is the velocity vector associated with the volume change of the precursor particles, and $R_{\mathrm{H_2O}}$ indicates the surface reaction of water. The diffusivity of water is assumed to be a function of temperature (T) according to the Arrhenius relation:

$$D_{\mathrm{H_2O}} = D_{0,\mathrm{diff,H_2O}} \exp\left(-\frac{Q_{\mathrm{v,H_2O}}}{R_{\mathrm{g}} T}\right) \tag{16}$$

Here, $D_{0,\mathrm{diff},\mathrm{H_2O}}$ is the pre-exponential factor of diffusivity, and $Q_{\mathrm{v},\mathrm{H_2O}}$ indicates the activation energy associated with the diffusion of water within cathode. The reactivity of water at the particle surface is given as,

$$R_{\mathrm{H_2O}} = \phi'(\rho) \cdot k_{\mathrm{reac},\mathrm{H_2O}} \cdot \tilde{c}_{\mathrm{H_2O}} \tag{17}$$

where, $\phi'(\rho)$ is an interpolation function, integral of which is already defined earlier in Eq. (11), that helps to identify the interfacial region, and $k_{\mathrm{reac},\mathrm{H_2O}}$ denotes the rate of removal of water molecules from the surface of the cathode precursor particles, which is defined by the Arrhenius relation as,

$$k_{\mathrm{reac},\mathrm{H_2O}} = k_{0,\mathrm{reac},\mathrm{H_2O}} \exp\left(-\frac{E_{\mathrm{reac},\mathrm{H_2O}}}{R_{\mathrm{g}}T}\right) \tag{18}$$

Here, $k_{0,\mathrm{reac},\mathrm{H_2O}}$ indicates the reference reaction rate coefficient and $E_{\mathrm{reac},\mathrm{H_2O}}$ is the activation energy for removal of water at the surface of the cathode particles. Since removal of water is being simulated here, if the concentration of water at the particle surface becomes zero, the rate of reaction is also expected to become zero, which justifies the presence of the normalized water concentration term $(\tilde{c}_{\mathrm{H_2O}})$ in the overall reaction rate (as shown in Eq. (17)). All the parameter associated with this water removal process is provided within the list of parameters included later.

Oxygen diffusion process: In order to simulate the diffusion of oxygen within the NMC particles, a multiphase diffusion equation is solved as shown below:

$$\frac{\partial \tilde{c}_{\mathrm{O_2}}}{\partial t} = \vec{\nabla} \cdot \left(D_{\mathrm{O_2}} \vec{\nabla} \mu_{\mathrm{O_2}}\right) - \vec{\nabla} \cdot \left(\tilde{c}_{\mathrm{O_2}} \vec{v}\right) + R_{\mathrm{O_2}} \tag{19}$$

$$\mu_{\mathrm{O_2}} = \delta F_{\mathrm{O_2}} / \delta \tilde{c}_{\mathrm{O_2}} \tag{20}$$

Here, $\tilde{c}_{O_2}(= c_{O_2}/c_{O_2,\max})$ is the normalized oxygen concentration within the cathode precursors, D_{O_2} is the diffusion coefficient of oxygen atoms within the cathodes, $\vec{v}$ is the velocity vector indicating bulk motion of the solid phase due to mechanical volume change, μ_{O_2} is the chemical potential of oxygen, R_{O_2} indicates the reaction for oxygen at the surface of the cathode particles, and F_{O_2} is the total free energy of oxygen within the cathode. Detailed expression of the total free energy F_{O_2} is provided below:

$$F_{O_2} = \int \left\{ f_{O_2}(\tilde{c}_{O_2}) + \frac{1}{2}\beta_{O_2}\left|\vec{\nabla}\tilde{c}_{O_2}\right|^2 \right\} dv \quad (21)$$

$$f_{O_2}(\tilde{c}_{O_2}) = A_{O_2}\left(\tilde{c}_{O_2} - c_{a,O_2}\right)^2 \cdot \left(\tilde{c}_{O_2} - c_{b,O_2}\right)^2 \cdot \left(\tilde{c}_{O_2} - c_{c,O_2}\right)^2 \quad (22)$$

Here, $f_{O_2}(\tilde{c}_{O_2})$ is the chemical free energy of oxygen within the cathode particles, for which the detailed expression is provided in Eq. (18), β_{O_2} is the gradient energy coefficient and A_{O_2}, c_{a,O_2}, c_{b,O_2} and c_{c,O_2} are some constants, detailed values of which will be provided within the list of parameters. The three different phases of oxygen concentration corresponds to the rocksalt (NiMn9505)O phase obtained right after the complete dehydration step, partially oxidized $(NiMn9505)_3O_4$ phase considered to be a mixture of (NiMn9505)O and $(NiMn9505)_2O_3$, and the completely oxidized $(NiMn9505)O_2$ phase. For these three different phases the concentrations of the second oxygen are 0.0, 0.33 and 1.0, respectively, which is adopted as the values for the c_{a,O_2}, c_{b,O_2} and c_{c,O_2} parameters. Even though the appearance of these exact phases is debatable, the developed computational technique provides a detailed framework that can be extended for more detailed and accurate analysis of the oxygen diffusion process within the cathode particles during the synthesis process. The magnitude of A_{O_2} and β_{O_2} (in Eqs. (21) and (22)) depends on the

surface energy of oxygen (γ_{O_2}) and length of the interfacial region (l_{O_2}) through the following equations:

$$A_{O_2} = 12\gamma_{O_2}/l_{O_2}, \text{ and, } \beta_{O_2} = 3\gamma_{O_2}l_{O_2}/2 \quad (23)$$

The reaction of oxygen at the surface is given as,

$$R_{O_2} = \phi'(\rho) \cdot k_{\text{reac},O_2} \cdot \left(1 - \tilde{c}_{O_2}\right) \quad (24)$$

where, $\phi'(\rho)$ is an interpolation function, integral of which is already defined earlier in Eq. (11), and k_{reac,O_2} indicates the rate of reaction, which is defined by the Arrhenius relation as,

$$k_{\text{reac},O_2} = k_{0,\text{reac},O_2} \exp\left(-\frac{E_{\text{reac},O_2}}{R_g T}\right) \quad (25)$$

Here, k_{0,reac,O_2} is the reference reaction rate coefficient and E_{reac,O_2} is the activation energy for reaction with oxygen at the surface of the cathode particles. Similar to the rate of reaction, an Arrhenius relation is used to capture the temperature dependence of oxygen diffusivity within the cathode particles,

$$D_{O_2} = D_{0,\text{diff},O_2} \exp\left(-\frac{Q_{v,O_2}}{R_g T}\right) \quad (26)$$

Here, D_{0,diff,O_2} is the pre-exponential factor and Q_{v,O_2} indicates the activation energy associated with the diffusion of oxygen within the cathode particles. Detailed list of parameters is provided later where the values of each of the constants are mentioned.

Lithium diffusion process: A solid solution type diffusion of lithium is assumed within the cathode particles. The following equation obtained from the Fick's second law is used for capturing the transport of lithium within the cathodes:

$$\frac{\partial \tilde{c}_{\mathrm{Li}}}{\partial t} = \vec{\nabla} \cdot \left(D_{\mathrm{Li}} \vec{\nabla} \tilde{c}_{\mathrm{Li}}\right) - \vec{\nabla} \cdot \left(\tilde{c}_{\mathrm{Li}} \vec{v}\right) + R_{\mathrm{Li}} \tag{27}$$

Here, $\tilde{c}_{\mathrm{Li}}\left(= c_{\mathrm{Li}}/c_{\mathrm{Li,max}}\right)$ is the normalized lithium concentration within the cathode precursors, D_{Li} is the coefficient of diffusivity for single phase diffusion of lithium within the cathodes, $\vec{v}$ is the velocity vector associated with the volume expansion and contraction experienced by the cathode precursor particles, and R_{Li} indicates the surface reaction of lithium. The diffusivity of lithium is assumed to be a function of temperature (T) according to the Arrhenius relation:

$$D_{\mathrm{Li}} = D_{0,\mathrm{diff,Li}} \exp\left(-\frac{Q_{\mathrm{v,Li}}}{R_{\mathrm{g}} T}\right) \tag{28}$$

Here, $D_{0,\mathrm{diff,Li}}$ is the pre-exponential factor of diffusivity and $Q_{\mathrm{v,Li}}$ indicates the activation energy associated with the diffusion of lithium within cathode. It is possible to have lithium concentration dependent diffusion coefficient to capture the variation in diffusivities in the densely-lithiated and sparsely-lithiated phases, but such a concentration dependence has not been taken into account in the present context. The reaction of lithium at the particle surface is given as,

$$R_{\mathrm{Li}} = \phi'(\rho) \cdot k_{\mathrm{reac,Li}} \cdot (1 - \tilde{c}_{\mathrm{Li}}) \cdot \tilde{c}_{\mathrm{O_2}} \tag{29}$$

where, $\phi'(\rho)$ is an interpolation function that demarcates the interfacial region where the chemical reaction occurs, integral of which is already defined earlier in Eq. (11), and $k_{\mathrm{reac,Li}}$ denotes the rate of reaction of the lithium source (LiOH in the present context) with the cathode particles, which is defined by the Arrhenius relation as,

$$k_{\mathrm{reac,Li}} = k_{0,\mathrm{reac,Li}} \exp\left(-\frac{E_{\mathrm{reac,Li}}}{R_{\mathrm{g}} T}\right) \tag{30}$$

Here, $k_{0,\mathrm{reac,Li}}$ indicates the reference reaction rate coefficient and $E_{\mathrm{reac,Li}}$ is the activation energy for reaction of lithium at the surface of the cathode particles. The dependence on oxygen

concentration in the lithium reaction term (see Eq. (30)) is consistent with the atomistic calculations, which clearly shows that presence of oxygen at the particle surface is necessary for initiating the lithiation process. Also, the lithium concentration is assumed to be capped by the oxygen level, because lithiation without oxygen is not possible as demonstrated by the atomistic calculations. All the parameter associated with this reaction of Li will be provided within the list of parameters included later.

Conversion from lithiated disordered rocksalt phase to the layered phase: Evolution of the layered phase within the lithiated disordered rocksalt structure occurs through two possible pathways:

a) Direct conversion from the disordered rocksalt to the layered phase, which can possibly be captured through a bulk chemical reaction term.
b) Growth of the layered phase which is mediated by the diffusion process.

Both direct conversion and diffusion induced layering is only possible if the lithium concentration is larger than that needed for the formation of the layered domains $\left(\tilde{c}_{\mathrm{Li}} > \tilde{c}_{\mathrm{Li,layer,cutoff}}\right)$. A solid solution type diffusion of the layered phase is assumed within the cathode particles. The following equation obtained from the Fick's second law is used for capturing the evolution and transport of the layered phase within the cathodes:

$$\frac{\partial \tilde{c}_{\mathrm{L}}}{\partial t} = \vec{\nabla} \cdot \left(D_{\mathrm{L}} \vec{\nabla} \tilde{c}_{\mathrm{L}}\right) - \vec{\nabla} \cdot \left(\tilde{c}_{\mathrm{L}} \vec{v}\right) + R_{\mathrm{L}} \quad (31)$$

Here, $\tilde{c}_{\mathrm{L}}$ indicates the fractional layering at a particular location within the cathode precursors, D_{L} is the coefficient of diffusivity experienced by the layered phase, $\vec{v}$ is the velocity vector associated with the volume expansion and contraction experienced by the cathode precursor particles, and R_{L} indicates the bulk reaction induced source term that governs the formation of the layered phase

from the disordered rocksalt one. The diffusivity of lithium is assumed to be a function of temperature (T) according to the Arrhenius relation:

$$D_{\mathrm{L}} = D_{0,\mathrm{diff,L}} \exp\left(-\frac{Q_{\mathrm{v,L}}}{R_{\mathrm{g}}T}\right) \cdot H\left(\tilde{c}_{\mathrm{Li}} - \tilde{c}_{\mathrm{Li,layer,cutoff}}\right) \tag{32}$$

Here, $D_{0,\mathrm{diff,L}}$ is the pre-exponential factor of diffusivity and $Q_{\mathrm{v,L}}$ indicates the activation energy associated with the diffusion of the layered phase within cathode. Also note that $H\left(\tilde{c}_{\mathrm{Li}} - \tilde{c}_{\mathrm{Li,layer,cutoff}}\right)$ is the Heaviside step function which controls the diffusion of the layered domains only where the normalized lithium concentration is greater than the cutoff lithium needed for the evolution of the layered phase $\left(\tilde{c}_{\mathrm{Li}} > \tilde{c}_{\mathrm{Li,layer,cutoff}}\right)$. The bulk reaction term that governs the evolution of the layered domains from the lithiated rocksalt one is given as:

$$R_{\mathrm{L}} = \phi(\rho) \cdot k_{\mathrm{reac,L}} \cdot \left(\left(\tilde{c}_{\mathrm{Li}}/\tilde{c}_{\mathrm{Li,layer,cutoff}}\right) - 1\right) \tag{33}$$

where, $\phi(\rho)$ is an interpolation function that demarcates the region interior to the cathode particles where the conversion from disordered rocksalt to the layered phase can occur, which is already defined earlier in Eq. (11), and $k_{\mathrm{reac,L}}$ denotes the rate of conversion from the lithiated rocksalt to the layered phase, which is defined by the Arrhenius relation as,

$$k_{\mathrm{reac,L}} = k_{0,\mathrm{reac,L}} \exp\left(-\frac{E_{\mathrm{reac,L}}}{R_{\mathrm{g}}T}\right) \tag{34}$$

Here, $k_{0,\mathrm{reac,L}}$ indicates the reference reaction rate coefficient and $E_{\mathrm{reac,L}}$ is the activation energy for conversion from lithiated rocksalt to layered domains within the bulk of the cathode particles. Direct conversion from the rocksalt to layered phase also depends on the supersaturation in lithium concentration which is defined by the ratio of $\tilde{c}_{\mathrm{Li}}/\tilde{c}_{\mathrm{Li,layer,cutoff}}$. All the parameter associated with this evolution of the layered domains will be provided within the list of parameters included later.

Volume expansion and mechanical stress generation: As the NiMn9505(OH)$_2$ precursors convert into the lithiated-NiMn9505-oxides (LiNiMn9505O$_2$), molar volume of the material changes during the entire conversion process. As a result, the precursor particles experience variation of species concentration induced volume contraction and/or expansion. This contraction/expansion leads to stress generation within the cathode particles, and under most of the cases this developed stress exceeds the yield limit and leads to viscoplastic deformation. In order to estimate the magnitude of mechanical stress and the increase in particle size due to volume expansion, the following equilibrium equations are solved:

$$\vec{\nabla} \cdot \boldsymbol{\sigma} = 0 \tag{35}$$

where, $\vec{\nabla}$ is the gradient operator, and $\boldsymbol{\sigma}$ indicates the mechanical stress tensor. Magnitude of stress is directly proportional to the elastic strain tensor $\left(\boldsymbol{\epsilon^{el}}\right)$ and the constant of proportionality $\left(\boldsymbol{C^{el}}\right)$, which is characterized as the stiffness matrix:

$$\boldsymbol{\sigma} = \boldsymbol{C}^{\mathbf{el}} : \boldsymbol{\epsilon}^{\mathbf{el}} \tag{36}$$

Under the assumption of small deformation, the total strain ($\boldsymbol{\epsilon}^{\mathbf{tot}}$) is divided into the elastic strain, species concentration induced chemical strain $\left(\boldsymbol{\epsilon}^{\mathbf{chem}}\right)$, and viscoplastic strain ($\boldsymbol{\epsilon}^{\mathbf{vp}}$), in an additive fashion:

$$\boldsymbol{\epsilon}^{\mathbf{tot}} = \boldsymbol{\epsilon}^{\mathbf{el}} + \boldsymbol{\epsilon}^{\mathbf{chem}} + \boldsymbol{\epsilon}^{\mathbf{vp}} \tag{37}$$

Change in the concentration of different species leads to expansion and/or contraction of the lattice volume of the transition metal based cathode precursors, which eventually results in a volumetric strain experienced by the cathode active particles. The species concentration induced volume change is assumed to give rise to a hydrostatic strain and depends on the partial molar volume of

the transition metal hydroxides $\left(\Omega_{\mathrm{TM(OH)_2}}\right)$, transition metal oxides (Ω_{TMO}), oxidized transition metal oxides $\left(\Omega_{\mathrm{TMO_2}}\right)$, and finally the lithiated transition metal oxides $\left(\Omega_{\mathrm{LiTMO_2}}\right)$ within the cathode particles. The incremental chemical strain is defined as:

$$\Delta\epsilon_{\mathrm{ij}}^{\mathrm{chem}} = \frac{1}{3}\cdot\begin{pmatrix} \left(\Delta\tilde{c}_{\mathrm{H_2O}}\cdot c_{\mathrm{H_2O,max}}\cdot\left(\Omega_{\mathrm{TM(OH)_2}} - \Omega_{\mathrm{TMO}}\right)\right) + \\ \left(\Delta\tilde{c}_{\mathrm{O_2}}\cdot c_{\mathrm{O_2,max}}\cdot\left(\Omega_{\mathrm{TMO_2}} - \Omega_{\mathrm{TMO}}\right)\right) + \\ \left(\Delta\tilde{c}_{\mathrm{Li}}\cdot c_{\mathrm{Li,max}}\cdot\left(\Omega_{\mathrm{LiTMO_2}} - \Omega_{\mathrm{TMO_2}}\right)\right) \end{pmatrix}\cdot\delta_{\mathrm{ij}} \tag{38}$$

where, δ_{ij} indicates the Kronecker delta function, and $\Delta\tilde{c}_{\mathrm{H_2O}}$, $\Delta\tilde{c}_{\mathrm{O_2}}$, and $\Delta\tilde{c}_{\mathrm{Li}}$ denotes the increments in water, oxygen, and lithium concentration. Here, lithiation is allowed to occur only after oxidation of the cathode particles. Adoption of such a complicated expression for the volumetric strain is necessary because the variation of the concentration of multiple different species is simulated here, and each of them have their own influence on the lattice volume. Note that the exact magnitude of the molar volumes depends strongly on the distribution of transition metals within the cathode precursors, such as, NMC111, NMC811 or NiMn9505, should demonstrate different magnitudes of molar volumes for the hydroxides, oxides, and the lithiated phases. The viscoplastic strain rate depends on the shear stress acting on the material, which is given as:

$$\dot{\boldsymbol{\epsilon}}^{\mathbf{vp}} = \dot{\gamma}\cdot\frac{3}{2}\frac{\boldsymbol{s}}{q_{\mathrm{vm}}} \tag{39}$$

Here, $\dot{\gamma}$ is a multiplying factor (detailed expression provided later), $\boldsymbol{s}$ is the deviatoric stress tensor, and q_{vm} is the effective von Mises stress. The deviatoric stress is defined as:

$$s_{\mathrm{ij}} = \sigma_{\mathrm{ij}} - \frac{1}{3}\sigma_{\mathrm{kk}}\delta_{\mathrm{ij}} \tag{40}$$

Here, σ_{kk} indicates the hydrostatic stress, which is defined as:

$$\sigma_{\mathrm{kk}} = \sigma_{11} + \sigma_{22} + \sigma_{33} \tag{41}$$

The effective von Mises stress (q_{vm}) is defined as:

$$q_{\mathrm{vm}} = \sqrt{\frac{3}{2}\left[s_{11}^2 + s_{22}^2 + (2.0 \times s_{12}^2)\right]} \tag{42}$$

This von Mises stress term indicates a combined form of the shear components of the stress tensor. The total viscoplastic strain as a function of time is written as:

$$\boldsymbol{\epsilon}^{\mathbf{vp},\mathrm{n}+1} = \boldsymbol{\epsilon}^{\mathbf{vp},\mathrm{n}} + \dot{\boldsymbol{\epsilon}}^{\mathbf{vp}} \cdot \Delta t \tag{43}$$

where, $\boldsymbol{\epsilon}^{\mathbf{vp},\mathrm{n}+1}$ and $\boldsymbol{\epsilon}^{\mathbf{vp},\mathrm{n}}$ is the total viscoplastic strain at the next and the present time step, $\dot{\boldsymbol{\epsilon}}^{\mathbf{vp}}$ is the viscoplastic strain rate as shown in Eq. (31), and Δt is the incremental time. The detailed expression of the multiplying factor $\dot{\gamma}$ is given as:

$$\dot{\gamma} = \left(q_{\mathrm{vm}} - \sigma_{\mathrm{y}}\right)/G_{\mathrm{vp}} \tag{44}$$

Here, σ_{y} is the yield stress and G_{vp} is the viscoplastic modulus. Usually, G_{vp} is a function of temperature, however in the present context no temperature dependence is taken into consideration. The evolution of yield stress $\left(\sigma_{\mathrm{y}}\right)$ with time is governed by the magnitude of viscoplastic strain ($\boldsymbol{\epsilon}^{\mathbf{vp}}$):

$$\sigma_{\mathrm{y}} = \sigma_{\mathrm{y},0} + H\epsilon_{\mathrm{eq}}^{\mathrm{vp}} \tag{45}$$

where, $\sigma_{\mathrm{y},0}$ is the initial yield stress, H is the strain hardening modulus, and $\epsilon_{\mathrm{eq}}^{\mathrm{vp}}$ indicates the equivalent viscoplastic strain, which is given as,

$$\epsilon_{\mathrm{eq}}^{\mathrm{vp,n+1}} = \epsilon_{\mathrm{eq}}^{\mathrm{vp,n}} + \dot{\epsilon}_{\mathrm{eq}}^{\mathrm{vp}} \cdot \Delta t \tag{46}$$

Here, $\epsilon_{eq}^{vp,n+1}$ and $\epsilon_{eq}^{vp,n}$ indicates the equivalent viscoplastic strains at the next and the present time steps, respectively, and $\dot{\epsilon}_{eq}^{vp}$ indicates the rate of change of equivalent viscoplastic strain, which is defined as:

$$\dot{\epsilon}_{eq}^{vp} = \sqrt{\frac{2}{3}\left[\dot{\epsilon}_{11}^{vp^2} + \dot{\epsilon}_{22}^{vp^2} + \left(2.0 \times \dot{\epsilon}_{12}^{vp^2}\right)\right]} \quad (47)$$

In order to simplify the computational procedure, all the viscoplastic strains are assumed to depend on the stresses obtained at the previous step. The equilibrium displacement obtained from the mechanics solve is imported into the governing equations for sintering (Eqs. (1) and (2)), expression for water removal (Eq. (15)), oxygen diffusion (Eq. (19)), lithium diffusion (Eq. (27)), and layered phase growth model (Eq. (31)) as the velocity vector ($\vec{v}$) associated with the volume expansion of the solid phase.

TableS3. List of parameters used in the computational analysis.

Name	Symbol	Unit	Value	Reference
Surface energy for NiMn9505	γ_{sf}	J/m^2	1.0	[18]
Grain-boundary energy for NiMn9505	γ_{GB}	J/m^2	0.42	[19]
Interfacial width	l	nm	20.0	Assumed
Activation energy for sintering for rocksalt like transition metal oxides and initial hydroxide phases	$E_{a,0}$	kJ/mol	80.0	[20]
Extra activation energy for oxidized transition metal oxide particles	E_{a,O_2}	kJ/mol	30.0	Fitted
Extra activation energy for lithiated transition metal oxide particles	$E_{a,Li}$	kJ/mol	80.0	Fitted
Decrease in activation energy for metal diffusion within the layered phase	$E_{a,L}$	kJ/mol	30.0	Fitted
Pre-exponential factor for the volumetric diffusion coefficient	$D_{v,0}$	m^2/s	$1.16x10^{-18}$	[21]
Activation energy for water diffusion	Q_{v,H_2O}	kJ/mol	32.0	

Pre-exponential factor for the diffusion of water	$D_{0,\mathrm{diff,H_2O}}$	$\mathrm{m^2/s}$	1.53x10^{-14}	
Activation energy for the surface reactivity of water during removal	$E_{\mathrm{reac,H_2O}}$	$\mathrm{kJ/mol}$	80.0	Fitted
Activation energy for oxygen diffusion	$Q_{\mathrm{v,O_2}}$	$\mathrm{kJ/mol}$	32.0	[22]
Pre-exponential factor for oxygen diffusion	$D_{0,\mathrm{diff,O_2}}$	$\mathrm{m^2/s}$	1.0x10^{-18}	[22]
Activation energy for reaction of oxygen at the particle surface	$E_{\mathrm{reac,O_2}}$	$\mathrm{kJ/mol}$	10.0	Fitted
Activation energy for lithium diffusion	$Q_{\mathrm{v,Li}}$	$\mathrm{kJ/mol}$	32.0	[23]
Pre-exponential factor for lithium diffusion	$D_{0,\mathrm{diff,Li}}$	$\mathrm{m^2/s}$	1.53x10^{-17}	Assumed
Activation energy for reaction of lithium salt with transition metal particles	$E_{\mathrm{reac,Li}}$	$\mathrm{kJ/mol}$	20.0	Fitted
Activation energy for the diffusion of the layered phase	$Q_{\mathrm{v,L}}$	$\mathrm{kJ/mol}$	30.0	
Activation energy for the conversion of lithiated rocksalt to the layered domains	$E_{\mathrm{reac,L}}$	$\mathrm{kJ/mol}$	10.0	Fitted
Critical normalized lithium concentration needed for the conversion from the rocksalt to the layered phase	$\tilde{c}_{\mathrm{Li,layer,cutoff}}$	--	0.8	
Young's modulus of NiMn9505 cathode particles	YM_{NiMn9505}	GPa	200.0	[24]
Initial yield strength of NiMn9505 cathodes	$\sigma_{\mathrm{y,0}}$	GPa	3.0	[24]
Partial molar volume of transition metal hydroxides	$\Omega_{\mathrm{TM(OH)_2}}$	$\mathrm{m^3/mol}$	26.24x10^{-6}	
Partial molar volume of transition metal oxides	Ω_{TMO}	$\mathrm{m^3/mol}$	12.27x10^{-6}	
Partial molar volume of oxidized transition metal oxides	$\Omega_{\mathrm{TMO_2}}$	$\mathrm{m^3/mol}$	18.9x10^{-6}	
Partial molar volume of lithiated transition metal oxides	$\Omega_{\mathrm{LiTMO_2}}$	$\mathrm{m^3/mol}$	21.42x10^{-6}	

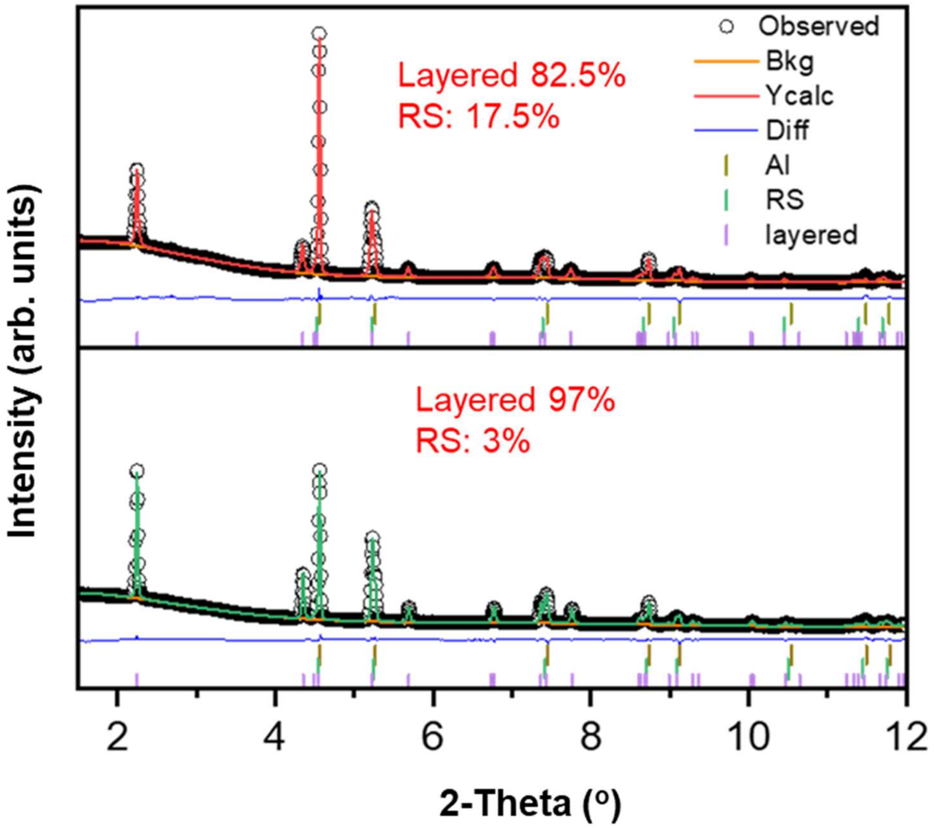

Fig. S18. The XRD patterns and Rietveld refinement of the NM9505-0.95Li and -1.05Li electrodes after 100 cycles.

References

1. Jain, A., et al., *Commentary: The Materials Project: A materials genome approach to accelerating materials innovation.* Apl Materials, 2013. **1**(1).
2. Loh, N.J., et al., *A review of two-step sintering for ceramics.* Ceramics International, 2016. **42**(11): p. 12556-12572.
3. Riewald, F., et al., *The LiNiO2 Cathode Active Material: A Comprehensive Study of Calcination Conditions and their Correlation with Physicochemical Properties Part II. Morphology.* Journal of The Electrochemical Society, 2022. **169**(2).
4. Wolfman, M., et al., *The Importance of Surface Oxygen for Lithiation and Morphology Evolution during Calcination of High‐Nickel NMC Cathodes.* Advanced Energy Materials, 2022. **12**(16).

5. Kotb, H.M., et al., *Sintering Temperature, Frequency, and Temperature Dependent Dielectric Properties of Na0.5Sm0.5Cu3Ti4O12 Ceramics.* Materials, 2021. **14**(17).
6. Park, H.J., et al., *Influence of sintering temperatures on microstructure and electrochemical performances of LiNi0.93Co0.04Al0.03O2 cathode for high energy lithium ion batteries.* Scientific Reports, 2022. **12**(1).
7. Chockalingam, K., et al., *2D Phase field modeling of sintering of silver nanoparticles.* Computer Methods in Applied Mechanics and Engineering, 2016. **312**: p. 492-508.
8. Clément, R.J., Z. Lun, and G. Ceder, *Cation-disordered rocksalt transition metal oxides and oxyfluorides for high energy lithium-ion cathodes.* Energy & Environmental Science, 2020. **13**(2): p. 345-373.
9. Van der Ven, A., J. Bhattacharya, and A.A. Belak, *Understanding Li Diffusion in Li-Intercalation Compounds.* Accounts of Chemical Research, 2013. **46**(5): p. 1216-1225.
10. German, R., P. Suri, and S. Park, *Review: liquid phase sintering.* Journal of Materials Science, 2009. **44**(1): p. 1-39.
11. German, R.M., *Liquid phase sintering*. 1985, New York: Plenum Press. xi, 240 p.
12. Wang, Y.U., *Computer modeling and simulation of solid-state sintering: A phase field approach.* Acta materialia, 2006. **54**(4): p. 953-961.
13. Chockalingam, K., et al., *2D Phase field modeling of sintering of silver nanoparticles.* Computer Methods in Applied Mechanics and Engineering, 2016. **312**: p. 492-508.
14. Suwa, Y. and Y. Saito, *Computer simulation of grain growth by the phase field model. Effect of interfacial energy on kinetics of grain growth.* Materials Transactions, 2003. **44**(11): p. 2245-2251.
15. Vanherpe, L., et al., *Bounding box algorithm for three-dimensional phase-field simulations of microstructural evolution in polycrystalline materials.* Physical Review E, 2007. **76**(5): p. 056702.
16. Ahmed, K., et al., *Phase field modeling of the effect of porosity on grain growth kinetics in polycrystalline ceramics.* Modelling and Simulation in Materials Science and Engineering, 2013. **21**(6): p. 065005.
17. Biswas, S., et al., *A study of the evolution of microstructure and consolidation kinetics during sintering using a phase field modeling based approach.* Extreme Mechanics Letters, 2016. **7**: p. 78-89.
18. Garcia, J.C., et al., *Surface structure, morphology, and stability of Li (Ni1/3Mn1/3Co1/3) O2 cathode material.* The Journal of Physical Chemistry C, 2017. **121**(15): p. 8290-8299.
19. Bojarski, S.A., M.P. Harmer, and G.S. Rohrer, *Influence of grain boundary energy on the nucleation of complexion transitions.* Scripta Materialia, 2014. **88**: p. 1-4.
20. Demirskyi, D., D. Agrawal, and A. Ragulya, *Neck growth kinetics during microwave sintering of nickel powder.* Journal of Alloys and Compounds, 2011. **509**(5): p. 1790-1795.
21. Pelleg, J., *Diffusion in ceramics*. 2016: Springer.
22. Ghosh, A., et al., *A Shrinking-Core Model for the Degradation of High-Nickel Cathodes (NMC811) in Li-Ion Batteries: Passivation Layer Growth and Oxygen Evolution.* Journal of The Electrochemical Society, 2021. **168**(2): p. 020509.
23. Kang, K. and G. Ceder, *Factors that affect Li mobility in layered lithium transition metal oxides.* Physical Review B, 2006. **74**(9): p. 094105.
24. Xu, R., et al., *Mechanical and structural degradation of LiNixMnyCozO2 cathode in Li-ion batteries: an experimental study.* Journal of The Electrochemical Society, 2017. **164**(13): p. A3333.
