# Supplementary material for: Cobalt-free composite-structured cathodes with lithium-stoichiometry control for sustainable lithium-ion batteries
Source: Nat Commun. 2024 Jan 10;15:430. doi: 10.1038/s41467-023-44583-3 (PMC10782004; doi:10.1038/s41467-023-44583-3)
Supplement: Supplementary file 3 — Description of Additional Supplementary Files [file 41467_2023_44583_MOESM3_ESM.pdf]

Description of Additional Supplementary Files

Supplementary Movie 1. 3D transmission X-ray microscopy (TXM) movie of NM9505-0.95Li with mixed Ni^{3+} and Ni^{2+}.

Supplementary Movie 2. 3DTXM movie of Ni^{2+} distribution.

Supplementary Movie 3. 3DTXM movie of Ni^{3+} distribution.
